# Targetable elements in SARS-CoV-2 S2 subunit for the design of pan-coronavirus fusion inhibitors and vaccines

**DOI:** 10.1038/s41392-023-01472-x

**Published:** 2023-05-10

**Authors:** Liyan Guo, Sheng Lin, Zimin Chen, Yu Cao, Bin He, Guangwen Lu

**Affiliations:** 1 Department of Emergency Medicine, State Key Laboratory of Biotherapy, West China Hospital, Sichuan University, Chengdu, Sichuan 610041 China; 2 Disaster Medicine Center, West China Hospital, Sichuan University, Chengdu, Sichuan 610041 China

**Keywords:** Vaccines, Target identification, Infectious diseases

## Abstract

The ongoing global pandemic of coronavirus disease 2019 (COVID-19), caused by severe acute respiratory syndrome coronavirus 2 (SARS‐CoV‐2), has caused devastating impacts on the public health and the global economy. Rapid viral antigenic evolution has led to the continual generation of new variants. Of special note is the recently expanding Omicron subvariants that are capable of immune evasion from most of the existing neutralizing antibodies (nAbs). This has posed new challenges for the prevention and treatment of COVID-19. Therefore, exploring broad-spectrum antiviral agents to combat the emerging variants is imperative. In sharp contrast to the massive accumulation of mutations within the SARS-CoV-2 receptor-binding domain (RBD), the S2 fusion subunit has remained highly conserved among variants. Hence, S2-based therapeutics may provide effective cross-protection against new SARS-CoV-2 variants. Here, we summarize the most recently developed broad-spectrum fusion inhibitors (e.g., nAbs, peptides, proteins, and small-molecule compounds) and candidate vaccines targeting the conserved elements in SARS-CoV-2 S2 subunit. The main focus includes all the targetable S2 elements, namely, the fusion peptide, stem helix, and heptad repeats 1 and 2 (HR1-HR2) bundle. Moreover, we provide a detailed summary of the characteristics and action-mechanisms for each class of cross-reactive fusion inhibitors, which should guide and promote future design of S2-based inhibitors and vaccines against new coronaviruses.

## Introduction

Coronaviruses (CoVs) are enveloped, positive-sense, single-stranded RNA viruses, classified in the family of *Coronaviridae*.^1^ They exist widely in nature and can infect humans, other mammals, or various avian species, causing different acute and chronic diseases.^2,3^ Coronaviruses are a highly diverse family comprising four genera: alphacoronavirus (α-CoV), betacoronavirus (β-CoV), gammacoronavirus (γ-CoV) and deltacoronavirus (δ-CoV).^4^ The four annually circulating human coronaviruses (HCoVs), namely HCoV-229E and HCoV-NL63 of α-CoV and HCoV-OC43 and HCoV-HKU1 of β-CoV, are endemic to humans and generally cause mild upper respiratory illness.^5–8^ However, there are also three highly pathogenic β-CoVs, including severe acute respiratory syndrome coronavirus (SARS-CoV), Middle East respiratory syndrome coronavirus (MERS-CoV), and severe acute respiratory syndrome coronavirus 2 (SARS‐CoV‐2).^9–11^ All three highly pathogenic HCoVs can cause severe pneumonia in humans and have led to numerous infections and deaths worldwide.^12–14^ The astonishingly high transmissibility of SARS-CoV-2 has resulted in a global coronavirus disease 2019 (COVID-19) pandemic that significantly impacted the global economy and public health, causing more than 763,740,140 confirmed infections and over 6,908,554 deaths as of 19 April 2023 (https://covid19.who.int).

Coronavirus infection begins with virus entry, which depends on the intact trimetric spike (S) protein located on the surface of the virion.^15–19^ The S protein, a class I viral fusion glycoprotein, is functionally divided into S1 and S2 subunits, which are responsible for receptor binding and membrane fusion, respectively. Specifically, S1 relies on receptor-binding domain (RBD) to interact with human angiotensin-converting enzyme 2 (ACE2).^16,20^ As a major antigenic determinant, RBD is a critical target for the development of vaccines and fusion inhibitors. Currently, most therapeutic neutralizing antibodies (nAbs) and promising vaccine candidates are designed to target the RBD or use RBD as the sole antigen.^21–25^ However, RBD is poorly conserved, with the newly emerged Omicron subvariants harboring multiple mutations in RBD that are capable of immune evasion from the majority of existing neutralizing antibodies (nAbs). Consequently, vaccine efficacy has markedly reduced, thus, posing new challenges for the prevention and treatment of SARS-CoV-2.^26–30^ Although efforts have been made to design nAbs and vaccines against Omicron subvariants, new variants might continue to emerge due to the progressive accumulation of mutations and the increasing immune selective pressure.^31^ Moreover, SARS-CoV-2 and MERS-CoV coinfection has been reported and may occur more frequently as the virus evolves.^32^ Given that β-CoVs infect the same target cells and use identical transcriptional regulatory sequences, genetic recombination between coronaviral coinfection in COVID-19 may result in new clades with higher transmissibility and lethality.^33^ Although many nAbs and vaccines against each class of β-CoVs have been developed with proven efficacy, they show weak cross-neutralization activity, thus, limiting their ability to prevent or treat highly pathogenic new HCoV infections.^34,35^

Sarbecovirus belongs to the *Betacoronavirus* genus and includes SARS-CoV-2, SARS-CoV, and their-related zoonotic coronaviruses isolated from bats, pangolins, etc., that share common features with SARS-CoV or SARS-CoV-2.^36,37^ These zoonotic coronaviruses may use multiple ACE2 orthologues for host cell entry and exhibit a similar binding affinity as the SARS-CoV-2 RBD-ACE2 interactions.^38–41^ Along with viral evolution, these zoonotic coronaviruses may, via cross-species transmission, lead to human infection and a potential pandemic. Although many nAbs against SARS-CoV-2 and SARS-CoV have been developed with proven efficacy, they exhibit weak cross-neutralizing activity against zoonotic coronaviruses. Hence, a need exists to develop broad-spectrum antiviral agents against sarbecovirus to combat future threat of zoonotic coronaviruses.^42^

In contrast to the massive accumulation of mutations within the SARS-CoV-2 RBD, the S2 fusion subunit has remained highly conserved among variants.^14^ It may, therefore, represents an ideal target for the design of cross-protective fusion inhibitors and candidate vaccines against new coronaviruses. Accordingly, herein, we review the most recently developed broad-spectrum fusion inhibitors and candidate vaccines targeting the conserved elements in SARS-CoV-2 S2 subunit. The main focus includes all the targetable S2 elements, namely, the fusion peptide (FP), heptad repeats 1 and 2 (HR1-HR2) bundle, stem helix (SH), and the related S2-targeted antivirals (e.g., nAbs, peptides, proteins, small-molecular compounds, and vaccines). In addition, the characteristics, and action-mechanisms for each class of cross-reactive fusion inhibitors are summarized in detail.

## Structure and function of the SARS-CoV-2 S2 fusion subunit

**Fig. 1:**
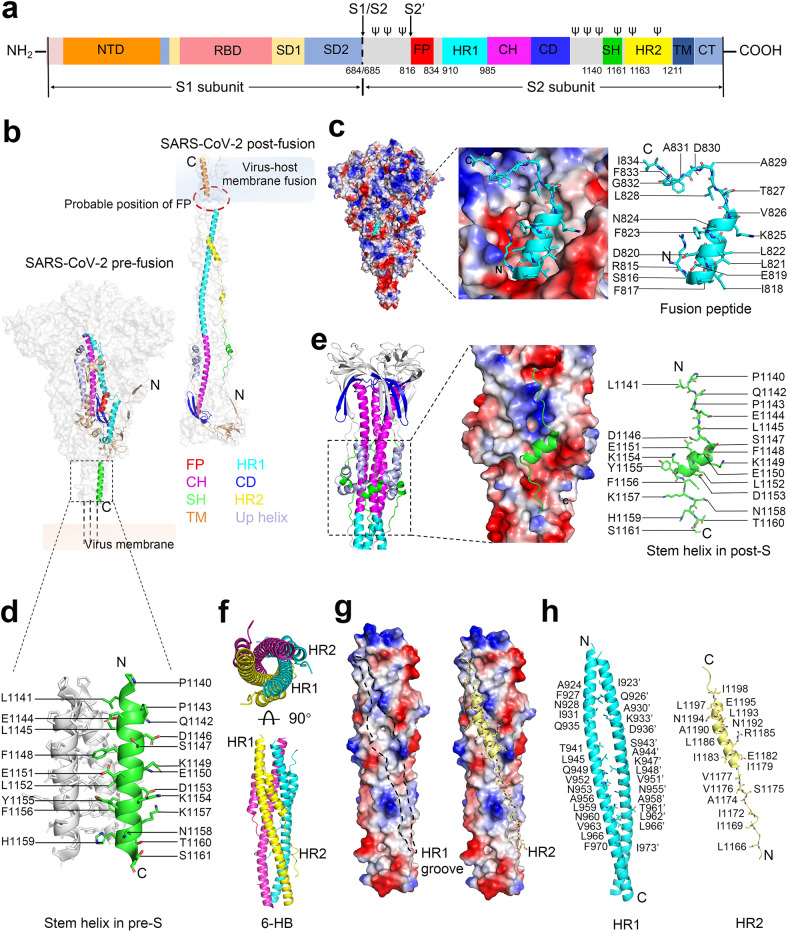
Structural features of targetable elements (fusion peptide, stem helix, and heptad repeats 1 and 2) in SARS-CoV-2 S2 subunit. **a** Schematic structure of SARS-CoV-2 spike protein. The conserved glycosylation sites within the SARS-CoV-2 S2 subunit are shown and marked as ψ. **b** Conformational transition of SARS-CoV-2 S2 subunit from pre- (PDB: 6XR8) to post-fusion (PDB: 7E9T) state. **c** The electrostatic surface representation of the FP binding area and the structural illustration of the FP in pre-fusion spike (PDB: 6XR8). The segment corresponding to FP is shown in sticks and colored in cyan. **d** The stem 3-helix bundle is shown in pre-S trimer (PDB: 6XR8). The detailed residues are shown in sticks and colored in green. **e** The electrostatic surface representation of the SH binding area and structural illustration of SH in post-fusion spike (PDB: 6XRA). The segment corresponding to SH is shown in sticks and colored in green. **f**–**h** Structure of the SARS-CoV-2 six-helix bundle (6-HB) in the post-fusion conformation (PDB: 6LXT). The symmetry-related structure of the 6-HB bundle is shown. Structures from a top view (up) and a side view (down) are presented in (**f**). The electrostatic surface of the HR2 binding site is shown in (**g**). The detailed hydrophobic and hydrogen-bond interactions between HR1 and HR2 are shown and labeled in (**h**). The distance cut-off is 3.1 Å for hydrogen-bond contact

Each of the three monomers is composed of 1273 amino acids (prototype) which constitute an S1 and S2 subunit heterodimer that form the bulbous head and stalk region, respectively (Fig. 1a). Topologically, the S N-terminus locates in the membrane-distal S1 subunit of the ecto-domain, and the short S C-terminus in the S2 subunit locates in the intracellular space (Fig. 1b).^43,44^ Structurally, the S1 subunit (the bulbous head) is divided into four domains: N-terminal domain (NTD), RBD, and two subdomains (SD1 and SD2), which wrap around the three-fold axis and shield the S2 subunit. The S2 subunit (the stalk) comprises various α-helical secondary structural motifs, including the FP, HR1, central helices (CH), connector domain (CD), SH, HR2, transmembrane domain (TM), and cytoplasmic tail (CT), which form a fusion-competent state after cleavage at the S2ʹ sites (Fig. 1a, b).^43,45^

The fusion process mediated by the SARS-CoV-2 S protein is similar to that of class I viral fusion proteins, such as influenza virus haemagglutinin (HA), human immunodeficiency virus (HIV) envelope, and mouse hepatitis virus S spike.^16,46^ Initially, the standing RBD will engage ACE2, and subsequently, S1 subunit will dissociate from S2, followed by the exposure of S2ʹ site and the cleavage of S2ʹ by the host proteases, releasing the structural constraints on the FP region. Membrane fusion could then occur along with a cascade of irreversible conformational transition events in the S2 subunit. HR1 undergoes a *“*jack-knife*”* refolding change to allow FP insertion into the host cell membrane. The folding back of the extended SH-HR2 element packs against the long central CH-HR1 coiled-coil, subsequently inducing the binding of SH to the outer region of CH and HR2 to the HR1 groove.^44,45^ Thus, the long central helical bundle formed by SH-HR2 with CH-HR1 is strengthened and becomes a very rigid fusion-core structure in S2, providing a strong force to hold the membrane together. In addition, this conformational change of S2 can induce the FP and the TM regions to co-locate at the same end to form the fusion pore in the post-fusion S state.^44,45^ Subsequently, membrane fusion occurs between viral particles and host cells, enabling the delivery of viral RNA into target cells. Therefore, S2 subunit is a crucial target for the design of antiviral drugs and vaccine candidates. Here, we summarize the detailed structural features of the targetable elements in pre-fusion or post-fusion SARS-CoV-2 S protein (Fig. 1b).

### Fusion peptide

SARS-CoV-2 S protein can be processed by the endopeptidase furin at the S1/S2 junction; subsequently, the cleaved S1 and S2 subunits remain noncovalently attached and fusion competent.^47^ Proteolytic cleavage at the S2ʹ site (R815, adjacent to FP) can be processed by enzymes like transmembrane protease serine 2 (TMPRSS2) via the plasma pathway or mediated by lysosomal cathepsins during virion endocytosis.^48^ As a result of S1 dissociation and S2′ site cleavage, a highly conserved short segment termed FP consisting mostly of hydrophobic residues spanning S816-I834 (residue numbering based on GenBank accession number of MN908947.3) is released and becomes inserted into the target cell membrane, thereby triggering the fusion event. Structurally, the buried FP can be divided into two segments: residues S_816_FIEDLLFNKV_826_ (residue numbering based on MN908947.3) in a short coiled-coil state located in a negative charged binding cavity and residues T_827_LADAGFI_834_ in a short loop state mostly positioned in a positively charged binding cavity (Fig. 1c).^44^

### Stem helix

The SARS-CoV-2 stem helix is composed of 22 amino acid residues spanning P_1140_LQPELDSFKEELDKYFKNHTS_1161_ (residue numbering based on MN908947.3), which forms a central triple-helix under the spike bundle to connect with the HR2 region (Fig. 1d).^44^ During conformational transition, a helical motif of the upstream helix (D737–A783, residue numbering based on MN908947.3) runs parallel to the CH groove via hydrophobic interactions, effectively forming a short six-helix bundle (hereafter named 6-HB-1) in post-S trimer (Fig. 1e).^44,45^ Due to the rotation of the connector domain, the extended SH-HR2 element aligns with the long-axis formed by the CH-HR1 domain, thus inducing the subsequent binding of SH to the outer region of the 6-HB-1, and HR2 to the hydrophobic groove formed by HR1.^44^ Specifically, a portion of SH (S_1147_FKEELDK_1154_, residue numbering based on MN908947.3) maintains the helical state, while the remaining segments of SH (P_1140_LQPELD_1146_ and Y_1155_FKNHTS_1161_, residue numbering based on MN908947.3) extend from the helix into a long loop to fit the extended post-fusion S2 state (Fig. 1e). The correct refolding of SH contributes to the formation of HR1-HR2 fusion bundle and the overall stability of the S2 fusion subunit, thus providing a promising therapeutic target for the design of fusion inhibitors and vaccines.^45^

### HR1-HR2 bundle

In pre-S, the buried HR1 (G910-D985, residue numbering based on MN908947.3) folds into four consecutive α-helices in the direction opposite to the downstream CH (Fig. 1b). During pre- to post-fusion transition, HR1 undergoes a *“*jack-knife*”* refolding to append to the CH, which forms an unusually long central three-stranded coiled coil (>180 Å) with three surface grooves between two adjacent HR1s. Subsequently, HR2 (D1163-K1211, residue numbering based on MN908947.3) binds to the hydrophobic HR1 grooves in an antiparallel manner to form HR1-HR2 6-HB fusion core by hydrophobic and hydrogen-bond interactions (Fig. 1f–h).^44,45^ Notably, the binding groove of HR1 forms and exposes in the intermediate state of S. Unlike HR1, HR2 is accessible in pre-S trimer.^49^ Overall, HR1 and HR2 can provide the promising therapeutic targets for the design of fusion inhibitors and vaccines.

### Coronavirus S2 subunit

**Fig. 2:**
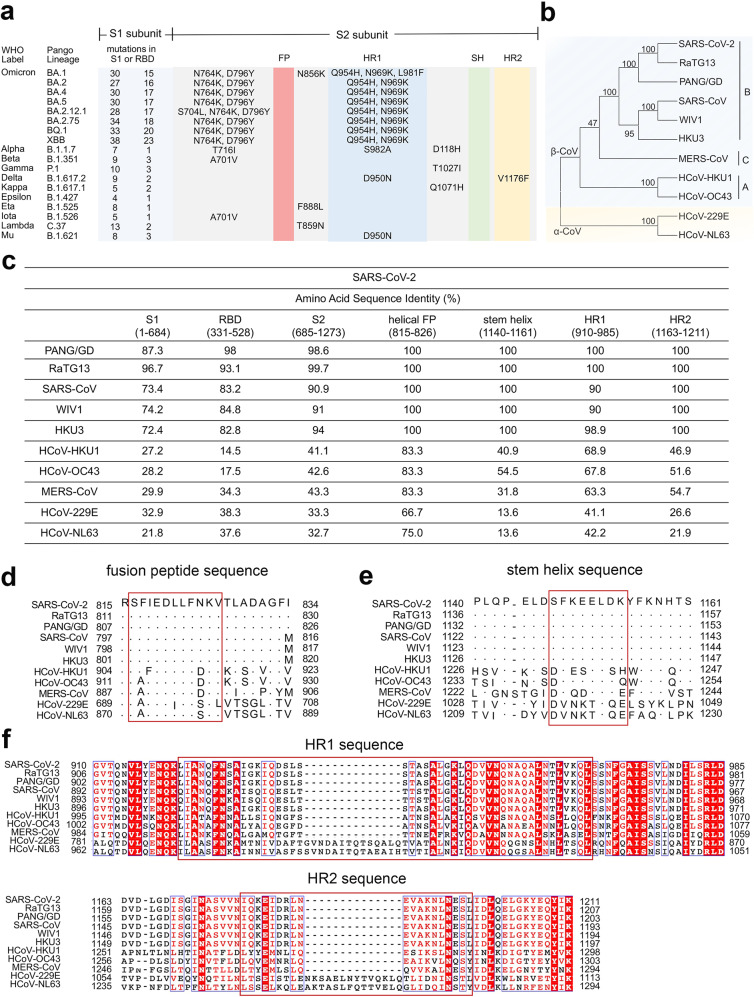
Sequence features of targetable elements (fusion peptide, stem helix, and heptad repeats 1 and 2) in coronavirus S2 subunit. **a** Amino acid mutations in the S2 subunit of SARS-CoV-2 variants (https://covariants.org). The number of mutations in S1 and RBD are shown. **b** Phylogenetic tree of typical coronaviruses, including SARS-CoV-2 (MN908947.3), RaTG13 (QHR63300.2), PANG/GD (QLR06867.1), SARS-CoV (AY278554.2), WIV1 (AGZ48828.1), HKU3 (QND76034.1), HCoV-HKU1 (YP_173238.1), HCoV-OC43 (NP_937950.1), MERS-CoV (QJX19948.1), HCoV-229E (NP_073551.1), and HCoV-NL63 (YP_003767.1), built via MEGA and maximum likelihood analysis of spike amino acid sequences. **c** Sequence identity of S and its targetable elements among SARS-CoV-2 and other coronaviruses. **d**–**f** Alignment of the fusion peptide (**d**) and stem helix (**e**), and HR1 and HR2 (**f**) among typical CoVs. The helical bundles of FP in the prefusion state and SH in the post-fusion state are outlined by a rectangle box. Residues composed of HR1-HR2 six-helical core are outlined. The dots represent similar residues, and the short lines represent missing residues

The sequence features of the targetable elements (e.g., the fusion peptide, stem helix, and HR1-HR2 bundle) in the S2 subunit of typical coronavirus, including SARS-CoV-2 variants, HCoVs, and various zoonotic coronaviruses, are shown in Fig. 2. The S2 subunit is more conserved among SARS-CoV-2 variants and β-CoVs, compared with RBD (Fig. 2a, c). The sequences of helical FP (Fig. 2c, d), SH (Fig. 2c and e), HR1 and HR2 (Fig. 2c, f) are highly conserved among sarbecoviruses, including the SARS-CoV-2 related lineage (SARS-CoV-2, its variants, Pangolin Guangdong 2019 (PANG/GD), and RaTG13) and the SARS-CoV related lineage (SARS-CoV, WIV1, and HKU3). In addition, the S2′ cleavage site (R815) remains unchanged among these typical coronaviruses (Fig. 2d). Moreover, the helical FP is also highly conserved among HCoVs (Fig. 2c, d), thus providing a promising cross-reactive target for pan-antivirals. Furthermore, HR1 and HR2 sequences shared the same length in β-CoVs (Fig. 2f). Notably, most mutations in the HR1 and HR2 of SARS-CoV-2 variants and other sarbecoviruses may not influence the global architecture of the post-fusion HR1-HR2 bundle,^50^ thus indicating that antiviral fusion inhibitors targeting the HR1-HR2 interface may exhibit effective cross-protection against the ongoing SARS-CoV-2 pandemic and the potential future outbreaks of other sarbecoviruses.

In conclusion, the structural rearrangement of coronavirus S2 subunit induces the formation of a stable post-fusion conformation, resulting in the fusion of viral envelope with the host cell membrane, and subsequent delivery of viral RNA into host cells. Due to the sequence homology of the S2 subunit among coronaviruses, it is likely that most coronaviruses share the same membrane fusion mechanism. Therefore, S2-based therapeutics can interfere with the formation of fusion machinery and provide cross-protection to combat infection with SARS-CoV-2 variants, other HCoVs, and zoonotic sarbecoviruses.

## Broad-spectrum therapeutic nAbs targeting the S2 fusion subunit

**Fig. 3:**
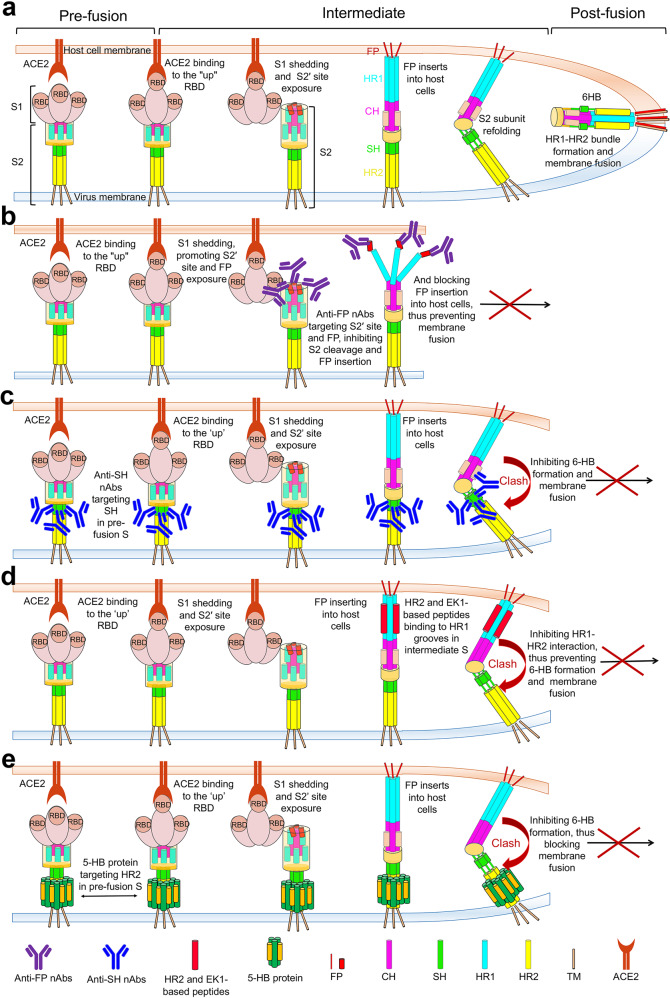
Proposed anti-viral mechanism for antibodies and fusion inhibitors targeting S2. **a** An overview of SARS-CoV-2 spike conformation change and its mediated membrane fusion. After the standing RBD engages ACE2 and S1 subunit dissociates, S2ʹ site will expose and be cleaved by the host proteases, followed by the insertion of FP into host cell membrane and the formation of HR1-HR2 six-helix bundle (6-HB). Subsequently, membrane fusion occurs between viral particles and host cells. **b**–**e** Proposed mechanism of virus neutralization by anti-FP nAbs (**b**), anti-SH nAbs (**c**), HR2 or EK1-based peptides/lipopeptides (**d**), and 5-HB proteins (**e**). Anti-FP nAbs bind to the uncovered S2ʹ site and FP after S1 dissociation, inhibiting S2ʹ cleavage and/or preventing FP insertion into the host cell membrane. Anti-SH nAbs bind to SH in pre-S, disrupting 6-HB formation to prevent membrane fusion. 5-HB proteins bind to HR2 in pre-S trimer and HR2/EK1-based peptides bind to the HR1 groove in the intermediate state of S, both of which would block 6-HB formation. FP fusion peptide, HR1 heptad repeats 1, CH central helix, SH stem helix, HR2 heptad repeats 2, TM transmembrane domain, CT cytoplasmic tail, nAbs neutralizing antibodies, HB helix bundle, ACE2 angiotensin-converting enzyme 2

**Fig. 4:**
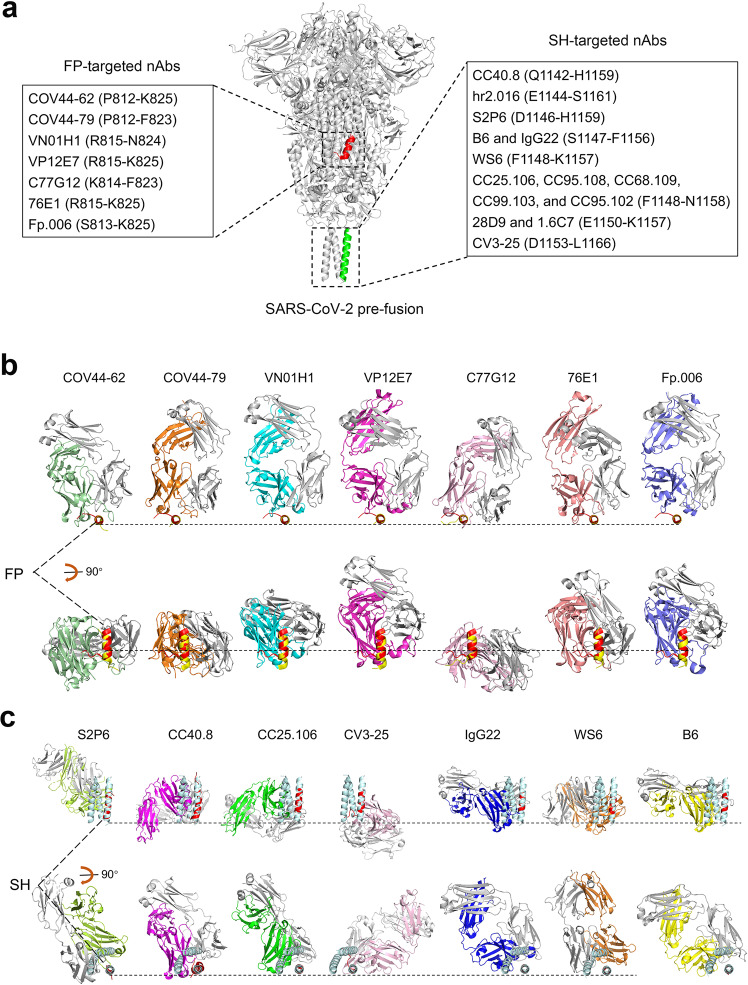
Broad-spectrum therapeutic nAbs targeting fusion peptide and stem helix. **a** S2 epitopes recognized by FP-targeted nAbs and SH-targeted nAbs are listed along with the SARS-CoV-2 pre-fusion S protein (PDB: 6XR8). Helical fusion peptide region (R815-V826) is colored red, and stem helix (P1140-S1161) is in green. **b**, **c** The binding mode of each nAb to fusion peptide (**b**) or stem helix (**c**). Each nAb-epitope peptide complex is aligned with the SARS-CoV-2 pre-fusion S protein (PDB: 6XR8) over fusion peptide (P812-V826_,_ red) or stem helix (P1140-S1161_,_ palecygan). The nAbs-bound peptides in the complex are colored yellow (**b**) and red (**c**), respectively. The light chain of each nAb is colored in gray, and the heavy chain is in a different color to distinguish

SARS-CoV-2 host-cell entry depends on S1 and S2 subunits, which mediate receptor binding in the pre-fusion conformation by S1 and induce membrane fusion along with the post-fusion change of S2. Thus, any antibody interfering with these processes may exhibit entry-inhibition activity (Fig. 3a). Most SARS-CoV-2 nAbs target the NTD or RBD of the S1 subunit, which may succumb to selective pressures and enhance immune escape as viral evolution. In contrast, recently reported nAbs targeting the FP or SH of the S2 subunit have shown cross-neutralizing activity against various coronaviruses (Fig. 4a).

### NAbs targeting a cryptic fusion peptide epitope

Fusion peptide is a targetable S2 element. NAbs that recognize the cryptic fusion peptide epitope could neutralize CoV infections (Fig. 3b). Currently, several FP-targeted monoclonal antibodies (mAbs) with broad neutralizing activity against CoVs have been isolated from COVID-19 convalescent patients, such as COV44-62, COV44-79, VN01H1, VP12E7, C77G12, 76E1, and fp.006 (Fig. 4a, b).^51–54^ The representative broad-spectrum nAbs targeting fusion peptide are summarized in Table 1.

**Table 1:** Representative cross-inhibitory nAbs targeting fusion peptide

Names	Sources	VH/L-class and CDR3-sequence	Binding epitope	Neutralizing action	Cell-cell fusion	IC50 values of pseudotyped coronaviruses (μg/ml)	IC50 values of live coronaviruses (μg/ml)	Protective efficacy	Refs.
COV44-62	COVID-19 convalescents	IGHV1-1: ASLLIVGGFDPLDDFEV, IGKV2-8: SSYGGTNNLL	P_812_SKRSFIEDLLFNK_825_ in FP	Binding to the uncovered S2ʹ site and FP after S1 dissociation, inhibiting S2ʹ cleavage and/or preventing FP insertion into host cell membrane	Inhibition of SARS-CoV-2 S-mediated membrane fusion	WT: 9.80, Alpha: 11.24, Beta: 7.06, Gamma: 12.80, Delta: 13.28, Mu: 6.52, Omicron-BA.1: 10.38, Omicron-BA.2: 20.26, SARS-CoV: 6.37, MERS-CoV: 25.48, HCoV-NL63: 1.06, HCoV-229E: 2.11	HCoV-OC43: 22.9	COV44-62 hardly limits SARS-CoV-2 infection in the Syrian hamster model	^51^
COV44-79		IGHV3-30: AILIIPGITEPGSPDALDI, IGKV1-12: QQASSFPWSIT	P_812_SKRSFIEDLLF_823_ in FP			WT: 21.53, Alpha: 24.21, Beta: 19.66, Gamma: 30.64, Delta: 45.33, Mu: 16.51, Omicron-BA.1: 33.02, Omicron-BA.2: 55.44, SARS-CoV: 20.91, HCoV-NL63: 3.24, HCoV-229E: 56.19, MERS-CoV>100	HCoV-OC43: 28.4	COV44-79 can limit SARS-CoV-2 infection in the Syrian hamster model	^51^
VN01H1	COVID-19 convalescents and vaccinated individuals	IGHV3-64: VKNSDVFRFPHLYFDV, IGKV3-15: QQYDNWPSIT	R_815_SFIEDLLFN_824_ in FP			WT: N.D., PDF-2180: 2.7 (in the absence of TMPRSS2); WT: 28.8, SARS-CoV: 16.1, MERS-CoV: 31.3, HCoV-NL63: 2.7, HCoV-229E: 2.5, WIV1: 0.4 (in the presence of TMPRSS2)	scFv format: WT: 7.3, BA.1: 0.9, BA.2: 2.6; IgG format: WT: 43.0, BA.1: 12.5, BA.2: 19.7	Preventing SARS-CoV-2 Gamma infection and reducing viral burden in the Syrian hamster	^52^
VP12E7		IGHV3-64: VKGLDVLRFLDLSTPSGERLDAFDI, IGKV1-9: QQLNSYPLFT	R_815_SFIEDLLFNK_825_ in FP			WT N.D., PDF-2180: 13.5 (in the absence of TMPRSS2); WT: 113.8, SARS-CoV: 24.1, MERS-CoV: 84.4, HCoV-NL63: 2.7, HCoV-229E: 13.6, WIV1: 8.0 (in the presence of TMPRSS2)	N.A	N.A	^52^
C77G12		IGHV3-30: ARGSDYVDDSPPLHY, IGKV1-16: QQYDSFPFT	K_814_RSFIEDLLF_823_ in FP			WT: 3.7, PDF-2180: N.D (in the absence of TMPRSS2); WT: 37.6, SARS-CoV: 37.6, MERS-CoV: 83.2, WIV1: 1.2, HCoV-NL63: N.D., HCoV-229E: N.D. (in the presence of TMPRSS2)	scFv format: WT: 2.0, BA.1: 0.6, BA.2: 0.8; IgG format: WT: 25.9, BA.1: 2.9, BA.2: 5.4	Preventing SARS-CoV-2 Gamma infection and reducing viral burden in the Syrian hamster	^52^
76E1	COVID-19 convalescents	IGHV3-43: AALVIVAAGDDFDL, IGKV3-43: CSYGGRNNLI	R_815_SFIEDLLFNK_825_ in FP			WT: 0.42, SARS-CoV: 4.8, MERS-CoV: 3.8, HCoV-229E: 1.2, HCoV-NL63: N.D., HCoV-OC43: N.D.	WT: 0.37, HCoV-229E: 1.6, HCoV-NL63: 3.9, HCoV-OC43: 4.6, SARS-CoV and MERS-CoV: N.D.	Preventing and treating SARS-CoV-2 and HCoV-OC43 infections in mice	^53^
Fp.006	COVID-19 convalescents	IGHV3-30: DLFLSDYDRSGYDPTRGGFDH, IGKV3-15: QQYGDWPLVT	S_813_KRSFIEDLLFNK_825_ in FP			WT: 0.7, Alpha: 15.6, Beta: 29.6, Gamma: 0.4, Delta: 50, Omicron-BA.1: 19.1, BA.2: N.D., BA.2.72: 80, BA.2.75.2: 30, BA.4/BA.5: N.D., WT: 3.1 (on cells with TMPRSS2)	N.A.	Preventing SARS-CoV-2 infection in mice	^54^

*N.D.*, indicates below neutralizing threshold. *N.A.,* not application. *scFv,* single-chain antibody fragment

COV44-62 and COV44-79 were the first identified anti-FP antibodies.^51^ They can broadly neutralize envelope pseudotyped viruses (PsVs) of CoVs, including SARS-CoV-2 with half maximal inhibitory concentration (IC50) values of 9.8 μg/mL for COV44-62 and 21.53 μg/mL for COV44-79, as well as its variant of concerns (VOCs) and the tested HCoVs.^51^ Mechanically, the complementarity determining regions (CDRs) on the heavy chains (H1, H2, H3) and light chains (L1 and L3) of COV44-62 interact with the P_812_SKRSFIEDLLFNK_825_ motif, while HCDR1, HCDR2, HCDR3, and LCDR3 of COV44-79 bind the P_812_SKRSFIEDLLF_823_ segment primarily via hydrogen bonds, salt bridges, and hydrophobic interactions.^51^ Furthermore, alanine mutation assays revealed that R815, E819, D820, L822, and F823 are the key residues recognized by COV44-62, and R815, E819, D820, and F823 are the most crucial for COV44-79 binding. Notably, a non-nAb, COV77-39, with a similar binding affinity to SARS-CoV-2 S, targets multiple residues around FP excluding R815, indicating that R815 at the S2′ cleavage site may be an indispensable recognition site for the design of anti-FP nAbs.^51^ However, compared with the COV44-62 Fc antibody, only the COV44-79 Fc antibody effectively limited SARS-CoV-2 infection in the Syrian hamster model. Furthermore, superimposition of the FP epitope structure bound by the two nAbs onto the intact SARS-CoV-2 pre-S conformation revealed that the FP epitope was buried and inaccessible, indicating that a conformational change or conformational dynamics around the FP segment may be a basic premise to accommodate these nAbs to bind FP.^51^

In addition, FP-targeted nAbs, VN01H1, VP12E7, and C77G12, isolated from COVID-19 convalescents and vaccinated individuals, bind a core epitope of FP spanning residues R_815_SFIEDLLFNK_825_.^52^ Specifically, VH01H1 and VP12E7 exhibit broad entry-inhibition activity against the tested α- and β-CoVs, in the presence of TMPRSS2.^52^ Compared to VH01H1 and VP12E7, C77G12 exerts an enhanced neutralization potency, however, only against β-CoVs, even in the absence of TMPRSS2. In addition, they can block SARS-CoV-2 S-mediated cell-cell fusion. Prophylactic administration of VN01H1 or C77G12 at high doses can reduce the pathology and burden of the SARS-CoV-2 Gamma (P.1) variant challenge in vivo, albeit with moderate potency.^52^ Structurally, these nAbs have slightly different binding modalities for the core epitope. Notably, the conserved R815 residue is concealed by the epitope/paratope interface of the three mAb Fabs via electrostatic interactions, indicating that these antibodies may neutralize SARS-CoV-2 infection by preventing S2′ cleavage, thereby impeding the conformational change of S2 subunit.^52^ Interestingly, ACE2-dependent enhancement of binding can be observed in these FP-specific nAbs, but not SH- or RBD-targeted nAbs (C21E3 and C94, respectively), suggesting that the cryptic FP epitope hidden in pre-S trimer may exposed following the conformational change induced by receptor binding.^52^

Recently, 76E1, a human antibody, also was isolated from COVID-19-recovered patients and exhibited broad neutralizing activity against various α- and β-CoVs.^53^ Moreover, 76E1 prevents SARS-CoV-2 and HCoV-OC43 infections in vivo. Similar to the FP-specific nAbs, 76E1 targets the helical FP epitope and the conserved S2′ site via forming a binding groove with H1, H2, H3, and L3 on the CDR loops of Fab. Moreover, receptor binding can induce the cryptic epitope to emerge, increasing the binding capacity of 76E1 to SARS-CoV-2 S protein.^53^ Notably, 76E1 retains antiviral activity during the viral post-attachment process. In contrast, the CB6 mAb targeting RBD does not, possibly because 76E1 binds to S after ACE2 binding, whereas CB6 competes with ACE2 for the RBD epitope.^24,53^

More recently, a FP-targeted nAb, fp.006, isolated from COVID-19 convalescents, binds to a core epitope (S_813_RRSFIEDLLFNK_825_) containing the S2ʹ site and the helical FP segment.^54^ Structural analysis further revealed that R815, E819 and F823 are the key residues recognized by fp.006, which is similar to FP-targeted nAbs (e.g., COV44-79 and COV44-79). The epitope peptide bound to fp.006 is folded as an amphipathic α-helix in the Fab groove.^54^ In addition, fp.006 cross-reacts with a variety of α- and β-CoV spike proteins and cross-inhibits pseudovirus infection of SARS-CoV-2 ancestral virus and its VOCs in vitro, and prevents SARS-CoV-2 infection in vivo, suggesting that it may be a promising antiviral drug candidate to combat the ongoing COVID-19 pandemic.^54^

Taken together, the highly conserved FP element, especially the short helical segment S_816_FIEDLLFNKV_826_, allows FP-targeted antibody to exhibit broad neutralizing activity and to counter the viral escape. Moreover, R815, within the SARS-CoV-2 ‘fusion activation’ proteolytic site, is also important for antibody recognition and neutralization. It is probably that these FP-targeted nAbs will form steric hinderance to prevent S2ʹ cleavage and/or inhibit FP insertion into the host cell membrane. In addition, superimposition of the FP epitope structure bound by each anti-FP nAb onto the pre-fusion S structure has suggested that these anti-FP nAbs bind to the core epitope with slightly different binding modalities, and that they all bind to the unexposed inner face rather than to the helical region fully accessible in the prefusion structure (Fig. 4b). These nAbs therefore are expected to bind the FP epitope after the exposure of the FP inner face (e.g., in the intermediate state of S). Accordingly, combined application of FP-targeted nAbs with RBD-specific nAbs or ACE2-mimics that destabilize pre-S is shown to enhance the therapeutic efficacy of antibodies against SARS-CoV-2.^52,53^

### NAbs targeting a vulnerable stem helix region

The stem helix is also a targetable S2 element (Fig. 3c). To date, several anti-SH nAbs with the different degree of cross-reactivity and cross-neutralizing activity against CoVs have been isolated from COVID-19 convalescent patients (S2P6, CC40.8, CV3-25, hr2.016, CC25.106, CC95.108, CC68.109, CC99.103, and CC95.102) or elicited by immunization in mice (WS6, B6, 1.6C7, 28D9, IgG22, and G5) (Fig. 4a, b and Table 2).^54–63^

**Table 2:** Representative cross-inhibitory nAbs targeting stem helix

Names	Sources	V-class and CDR3-sequence	Binding epitope	Neutralizing mechanism	Binding affinity to viral S protein (nM)	S-mediated cell-cell fusion	IC50s of pseudotyped coronaviruses (μg/ml)	IC50s of live coronaviruses (μg/ml)	Protective efficacy	Refs
S2P6	COVID-19 convalescent donors	IGHV1-46: ARGSPKGAFDY, IGKV3-20: QQYGSSPPRFT	D_1146_SFKEELDKYFKNH_1159_	Disturbing stem helical bundle, blocking 6-HB formation, and preventing virus-host membrane fusion	SARS-CoV-2: 7, SARS-CoV: 7, MERS-CoV: 12, HCoV-OC43: 16, HKU1:~120	Inhibition SARS-CoV-2 S-mediated membrane fusion	SARS-CoV-2: 1.4, SARS-CoV: 2.4, MERS-CoV: 17.1, HCoV-OC43: 1.3, PANG/GD: 0.02; Gamma, Alpha, Beta, and Kappa: 0.7~3.0	SARS-CoV-2: 1.67 (in VeroE6-TMPRSS2 cells)	Prophylactic use of S2P6 can reduce viral burden in the Syrian hamster challenged with SARS-CoV-2	^55^
CC40.8		IGHV3-23: AITMAPVV, IGKV3-10: YSTDSSGNHAV	Q_1142_PELDSFKEELDKYFKNH_1159_		SARS-CoV-2 < 0.001		WT, Gamma, Alpha, Beta, and Delta: 6.0~13.0; SARS-CoV: 15.0; SHC014: 1.0; PANG: 14.0, WIV1: 6.0, MERS-CoV>300	N.A.	Prophylactic use of CC40.8 can reduce viral burden in mice and Syrian hamster challenged with SARS-CoV-2	^56^
CV3-25		IGHV5-51: LPQYCSNGVCQRWFDP, IGKV1-12: QQGNSFPYT	D_1153_KYFKNHTSPDVDL_1166_		SARS-CoV-2: 0.6	N.A.	WT, Alpha, Beta, Delta, Gamma, Omicron, WIV1, and SARS-CoV: ~0.1 to ~0.8	N.A.	N.A.	^57^
hr2.016		IGHV3-23: AKLLVVFVIDY, IGKV3-20: QQYGSSPPT	E_1144_LDSFKEELDKYFKNHTS_1161_		SARS-CoV-2 (e.g., WT, Delta, Omicron BA.1, and BA.4/5) and MERS-CoV: 29.3 to 104.7 ng/mL	N.A.	WT: 0.01, Alpha: 0.3, Beta: ~0.5, Gamma: ~0.6×10^−3^, Delta: 0.3, Omicron-BA.1: ~0.5, BA.2: ~0.6, BA.2.72: ~1.1, BA.2.75.2: ~0.9, BA.4/BA.5: ~0.9, WT: 1.2×10^−2^ (on cells with TMPRSS2)	N.A.	Preventing SARS-CoV-2 infection in mice	^54^
CC25.106	COVID-19 convalescent-vaccinated donors	IGHV1-46: ARGGVHGLDY, IGKV1-51: GTWDTNLGAFV	F_1148_KEELDKYFKN_1158_		SARS-CoV-2: 0.98, MERS-CoV: 0.59	N.A.	WT: 3.8, SARS-CoV: 12, WIV1: 16, PANG17: 36, SHC014: 0.8, MERS-CoV: 3.3, Alpha: 9.3, Beta: 27, Gamma: 20, Delta: 25, Various Omicron subvariants: 14~43	N.A.	Preventing SARS-CoV-2, SARS-CoV, and MERS-CoV infections in mice	^58^
CC95.108		IGHV1-46: ARDSRGPGIF, IGKV1-51: GAWDSTPGTWV			SARS-CoV-2: 0.63, MERS-CoV: 1.79	N.A.	WT: 1.5, SARS-CoV: 7.5, WIV1: 7.1, PANG17: 13, SHC014: 0.5, MERS-CoV: 0.5, Alpha: 2.8, Beta: 5.9, Gamma: 2.1, Delta: 6.9, Omicron subvariants: 4.3~12	N.A.	N.A.	
CC68.109		IGHV1-46: ARGSNWGPWDY, IGKV3-20: QQYDSSPPIFT			SARS-CoV-2: 1.15, MERS-CoV: 8.8	N.A.	WT: 2.0, SARS-CoV: 14, WIV1: 18, PANG17: 35, SHC014: 1.6, MERS-CoV: 2.3	N.A.	Preventing SARS-CoV-2, SARS-CoV, and MERS-CoV infections in mice	
CC99.103		IGHV1-46: ASGILTGLFDY, IGKV3-20: LQYGSSPPIFT			SARS-CoV-2: 0.43, MERS-CoV: 3.2	N.A.	WT: 5.1, SARS-CoV: 4.4, WIV1: 6.3, PANG17: 12, SHC014: 0.3, MERS-CoV: 1.5, Alpha: 5.8, Beta: 12, Gamma: 14, Delta: 12, Omicron subvariants: 7.5~20	N.A.	Preventing SARS-CoV-2, SARS-CoV, and MERS-CoV infections in mice	
CC95.102		IGHV1-46: ARDSRGPGIF, IGKV1-51: GAWDSTPGTWV			MERS-CoV: 3.2	N.A.	WT: 0.1, SARS-CoV: 1.0, WIV1: 0.9, PANG17: 1.3, SHC014: 0.1 MERS-CoV: >300, Alpha: 0.3, Beta: 0.8, Gamma: 0.2, Delta: 0.4, Omicron subvariants: 0.7~1.8	N.A.	N.A.	
WS6	Mice immunized by SARS-CoV-2 S mRNA	IGHV1-5: TRTGSYFDY, IGKV4-61: QQYQSYPPT	F_1148_KEELDKYFK_1157_		N.A	N.A.	WT, Alpha, Beta, Gamma, Delta, Mu, and Omicron: 2.46~26.52; RaTG13, PANG/GD, PANG/GX, SARS-CoV, WIV1, and SHC014: 0.11~4.91; MERS-CoV: N.D.	N.A.	N.A.	^59^
B6	Mice co-immunized with MERS-CoV S and SARS-CoV S antigens	IGHV1-19: ARQLGRGNGLDYW, IGKV8-27: HQYLSSYT	S_1147_FKEELDKYF_1156_		MESR-CoV: 10^-3^, HCoV-OC43: 10^-2^, SARS-CoV: 1.0, SARS-CoV-2: 1.0	N.A.	MERS-CoV: 1.7; HCoV-OC43: 4.0; HKU4: 2.4; SARS-CoV and SARS-CoV-2: N.D.	N.A.	N.A.	^60^
28D9	Humanized mice co-immunized with S antigens from MERS-CoV, SARS-CoV and HCoV-43	IGHV6-1: ARVPMNRGGMDV, IGKV4-1: HQYYSIPNT	E_1150_ELDKYFK_1157_		N.A.	Inhibition MERS-CoV S-mediated membrane fusion	MERS-CoV: 0.13, SARS-CoV: 60.5, SARS-CoV-2: 45.3, HCoV-OC43: 64.9	MERS-CoV: 0.93, SARS-CoV-2 and SARS-CoV: N.D.	N.A.	^61^
1.6C7		IGHV6-1: ARATLARGALDY, IGKV4-1: QQYYSTPWT			N.A.		MERS-CoV: 0.39, SARS-CoV>300, SARS-CoV-2 > 300, HCoV-OC43 > 300	MERS-CoV: 0.08, SARS-CoV-2 and SARS-CoV: N.D.	N.A.	
IgG22	MERS-CoV spike stabilized stem antigens immunized mice	IGHV1-19: VRGNDYHGRAMDY, IGKV1-99: FQSNYLFT	S_1147_FKEELDKYF_1156_		Fab format: MERS-CoV: 2.9, SARS-CoV: 7.2, SARS-CoV-2: 6.7	N.A.	N.A.	MERS-CoV: 0.12, SARS-CoV-2: N.D.	Prophylactic use of IgG22 can prevent MERS-CoV and SARS-CoV-2 infections in mice	^62^

*WT,* wild type (of SARS-CoV-2). *N.D.*, indicates below neutralizing threshold. *N.A*., not application

#### Anti-stem helix nAbs isolated from COVID-19 convalescent patients

S2P6, identified by Pinto et al., can cross-react with S proteins of human-infected β-CoVs.^55^ Importantly, S2P6 exhibits high neutralizing potency against PsVs of SARS-CoV-2 and its variants, as well as the tested HCoVs and PANG/GD. Moreover, the Fc-mediated effector functions of S2P6 can enhance protection against SARS-CoV-2 infection in vivo.^55^ Structural analysis further revealed that the epitope peptide bound to S2P6 is folded as an amphipathic α-helix and spans D_1146_SFKEELDKYFKNH_1159_ via shape-complementarity and hydrogen bonding. In addition, the hydrophobic side of the epitope peptide interacts with S2P6 via residues F1148, L1152, Y1155, and F1156 with S2P6 antibody’s HCDR1, HCDR2, and HCDR3, as well as LCDR1 and LCDR3.^55^ Mechanistically, S2P6 binding to viral S may disrupt the stem-helical bundles and sterically block SARS-CoV-2 S fusogenic rearrangements.^55^ Moreover, S2P6 binds to S protein with comparable affinities in pre- and post-fusion conformations, indicating that SH may be accessible in all S trimer conformations, unlike the FP epitope, which is accessible only in the intermediate conformation (Fig. 3b, c).^55^ Moreover, similar to that observed for FP-targeted nAbs (e.g., VH01H1 and VP12E7), S2P6 exhibits full neutralizing activity in the presence of TMPRSS2, however, is less effective in the absence of TMPRSS2, suggesting that their binding may be influenced by endosomal pH.^52,55^ Indeed, the interaction between S2P6 and S protein is pH-dependent, with the higher binding affinity at serological-pH than at endosomal-pH, indicating that its entry-inhibition capacity may be decreased when virus entering into host cells via the endosomal pathway relative to the membrane fusion route.^55^ Moreover, S2P6 does not compete with anti-FP nAbs, such as COV44-62 or COV44-79, for spike binding, indicating that a bispecific antibody that combines SH and PF epitopes might exhibit more potent antiviral neutralizing activity.^51,55^

CC40.8—another anti-SH antibody—exhibited broad neutralizing activity against sarbecoviruses from clades 1a (PANG, SARS-CoV-2, and its VOCs) and 1b (SARS-CoV, WIV1, and SHC014).^56^ Despite the relatively low neutralizing potency against SARS-CoV-2 PsVs, CC40.8 still exhibits potent protection against SARS-CoV-2 challenge in mice and hamster models.^56^ Structurally, the epitope residues targeted by CC40.8 also adopt a helical structure comprising residues Q_1142_PELDSFKEELDKYFKNH_1159_ which span the hydrophobic groove formed by the CC40.8 paratope. Among them, residues L1145, F1148, E1151, and Y1155 are the key contact epitopes for antibody binding and neutralization.^56^ Moreover, two possible neutralization mechanisms have been described for CC40.8, in which antibody binding may conflict with neighboring protomers, causing the stem-helical bundle fragmentation in the pre-fusion state and therefore promoting premature spike activation. Alternatively, antibody binding may clash with the post-fusion conformation when superimposed, thus, hindering the formation of the 6-HB fusion machinery.^56^ Notably, cross-reactive nAbs targeting the CC40.8 SH epitope can be infrequently induced following natural infection with HCoVs, relative to strain-specific nAb responses.^64^

Another mAb-CV3-25 can neutralize PsVs of SARS-CoV-2, its VOCs, as well as SARS-CoV and WIV1 in vitro, with IC50s values of 0.1 ~ 0.8 μg/mL.^57^ Unlike B-cell lineage-derived anti-SH mAbs, such as S2P6 and CC40.8, which bind to the hydrophobic surface of the amphipathic epitope, CV3-25 interacts with the relatively solvent-exposed hydrophilic surface in a distinct S2 stem-helix epitope that spans residues D_1153_KYFKNHTSPDVDL_1166_, thereby identifying an auxiliary vulnerability site on the pathogenic β-CoVs.^57^ Interestingly, CV3-2 binding to the epitope peptide almost exclusively depends on the heavy chain. In addition, the alignment of the CV3-25-SH structure to the molecular dynamic model of the full length S ectodomain suggests that the light chain of CV3-35 likely interacts with residues in the downstream region of SH and the initiation region of HR2, spanning D_1168_ISGINASVVN_1178_, which differs from other SH reignition mAbs.^57^

Nowadays, hr2.016 is probably the most effective cross-neutralizing human antibody of all identified SH-targeted nAbs against PsVs of SARS-CoV-2 (with an IC50 of 10 ng/ml) and the tested VOCs (with IC50s of 0.6 ~ 1082 ng/ml).^54^ Moreover, hr2.016 can effectively prevent SARS-CoV-2 infection in vivo. However, the complex structure of hr2.016-SH has not been clarified. Notably, ACE2-dependent enhancement of binding is observed in hr2.016-spike interactions (2.3-fold for SARS-CoV-2, and about 5-fold for the Omicron subvariants), suggesting that hr2.016 may recognize different epitopes in the S conformational change or through an induced fit mechanism at the binding interface.^54^

Notably, Zhou et al. recently identified a large panel of cross-reactive nAbs from vaccinated individuals that recognize a common hydrophobic SH epitope (F_1148_KEELDKYFKN_1158_) in the S2 fusion subunit of β-CoVs.^58^ These nAbs show potent cross-neutralization against β-CoVs, including SARS-CoV-2, the tested VOCs, SARS-CoV, MERS-CoV, and various zoonotic sarbecoviruses. Among them, CC25.106, CC68.109 and CC99.103 can effectively provide cross-protection in mice challenged with the three HCoVs that cause deadly disease.^58^ Three hydrophobic residues, F1148, L1152, and F1156, are the most critical epitope residues for nAbs binding, which is the same as other reported anti-SH nAbs (e.g., S2P6).^58^ Moreover, they found that the heavy chains of these anti-SH nAbs were primarily derived from IGHV1-46 (63%) and IGHV3-23 (19%) germline gene families, while the light chains were encoded by IGKV3-20 (47%) and IGLV1-51 (16%) germline genes. Notably, the broad neutralizing SH-targeted antibodies, e.g., S2P6 and CC40.8, were also derived from IGHV1-46 and IGHV3-23 germline gene families, respectively. In addition, PPxF modification in CDR3 of the light chain was essential for the broadly neutralizing SH-targeted antibodies to neutralize multiple beta-coronaviruses. The structural analysis results showed that there are two SH-binding modes for IGHV1-46-encoded nAbs. The two modes were differentiated by the light chain residues. Overall, their results reveal the molecular basis of these anti-SH nAbs and enrich the common features of antibody germline-encoded residues that are vital for SH-targeting, which may guide and promote the design of the pan-betacoronavirus vaccines.^58^

SH-targeted antibodies can be evoked during natural infection. A dominant linear epitope SH-peptide, F_1148_KEELDKYFKNH_1159_, that is naturally recognized by serum antibodies in ~90% of COVID-19 convalescents has been identified by Li et al.^65^ In addition, Altin et al. suggested that peptide E_1150_ELDKYFK_1157_ is the minimal linear epitope of stem helix and is widely recognized in COVID-19 convalescence. Importantly, SARS-CoV-2 infection can induce antibodies to this epitope that can cross-react with the pandemic and endemic coronavirus S antigens.^66^ Moreover, a prior exposure to endemic coronaviruses (e.g., HCoV-OC43 and HCoV-229E) could induce a pre-existing immune response to the conserved SH epitope in non-COVID-19 exposed donors.^66^ This is further supported by the fact that a higher frequency of polyclonal nAbs targeting SH region may reduce disease severity in COVID-19 donors.^65,67^

#### Anti-stem helix nAbs from immunized mice

WS6 was isolated from a mouse immunized with SARS-CoV-2 S mRNA that can bind to SH (F_1148_KEELDKYFK_1157_).^59^ It can effectively prevent SARS-CoV-2, all the VOCs and other tested sarbecovirus infections. The neutralization mechanism of WS6 is similar to that of CC40.8. Moreover, residues F1148, L1152, Y1155, and F1156 are critical contact epitopes for WS6 binding and neutralization.^59^ In addition, Shi et al. compared the epitopes of six anti-SH nAbs (e.g., S2P6, CV3-25, IgG22, CC40.8, B6, and WS6) and subsequently identified a supersite of vulnerability.^59^ The majority of SH-targeted nAbs could recognize this supersite of the vulnerable segment, F_1148_KEELDKYF_1156_.^59^ This stem-helical supersite is highly conserved among sarbecoviruses, indicating a broad neutralization activity of anti-SH antibodies at this site.^59^

Other anti-SH nAbs (B6, 28D9, 1.6C7, and IgG22) were identified from mice immunized with MERS-CoV S antigen alone or in combination with SARS-CoV S and/or HCoV-OC43 S antigens.^60–62^ They can recognize a similar epitope in the SARS-CoV-2 SH (S_1147_FKEELDKYFK_1157_). Notably, they can cross-react with S proteins of most human-infected β-COVs (SARS-CoV-2, SARS-CoV, MERS-CoV, and HCoV-OC43) and neutralize infection against MERS-CoV PsV and/or authentic MERS-CoV. However, they do not exhibit entry-inhibition activity against authentic SARS-CoV-2 and/or SARS-CoV-2 PsV, except for 28D9 with a weak neutralizing activity against SARS-CoV-2 PsV with an IC50 value of 45.3 μg/mL.^60–62^ Notably, IgG22 can prevent MERS-CoV and SARS-CoV-2 challenges in vivo.^62^

Recently, a study reported that MERS-related bat coronaviruses, including NeoCoV and PDF-2180, can utilize specific bat ACE2 orthologues rather than Dipeptidyl peptidase 4 (DPP-4, the receptor of most merbecoviruses) as an entry receptor.^68^ Notably, the NeoCoV PsV containing a T510F RBD mutation could effectively enter into human ACE2 cells, suggesting a potential zoonotic threat to humans. Although anti-RBD nAbs for SARS-CoV-2 or MERS-CoV did not cross-neutralize PDF-2180 or NeoCoV, two broad-spectrum SH-targeted nAbs (e.g., S2P6 and B6) can effectively cross-inhibit PDF-2180, NeoCoV, and NeoCoV-T510F PsVs entry into Bat40ACE2- or human ACE2 cells.^68^ In addition, the sequence and structure of the helical FP between MERS-CoV and PDF-2180 are highly conserved, suggesting that FP-targeted nAbs may be also effective in preventing the potential PDF-2180 infection.^68^

It should be noted that the N-glycan site N1158 is present in SH. The SH epitopes of most nAbs (e.g., S2P6, CC40.8, CV3-25, Hr2.016, CC25.106, and CC95.108) (Fig. 4a) contain glycan residues at this site. Despite of the glycan decorations, these nAbs still exhibited potent neutralizing activities against SARS-CoV-2 prototype and its variants. Structurally, most anti-SH nAbs bind to the unexposed inner surface of the stem-helices (Fig. 4c), whereas N1158 residue in SH is solvent-exposed and may not directly participate in nAbs-SH interactions.^44,45^ The T1160A mutation could eliminate the AsnHisThr (NHT) N-glycosylation motif. The neutralization sensitivity of CC40.8 to the SARS-CoV-2 T1160A variant was only slightly increased as compared to the wild-type virus, indicating that the steric obstruction caused by N1158 glycan on SH region was limited.^56^

In conclusion, SH-targeted nAbs can be identified from COVID-19 convalescents or vaccinated animals. In addition, they are nearly all bind to the unexposed inner face rather than to the helical region fully accessible in the prefusion structure with different binding modes and varying orientations (Fig. 4c). These nAbs exert cross-neutralization by disrupting pre-fusion S conformation or sterically preventing 6-HB formation and blocking membrane fusion (Fig. 3c), suggesting they are promising candidate antibodies to combat the ongoing COVID-19 pandemic. In addition, the conserved SH epitopes are potential targets for the design of fusion inhibitors and next-generation vaccines against SARS-CoV-2 variants, novel recombinant coronaviruses, and potentially zoonotic coronaviruses. Drug combinations offer numerous advantages over monotherapies, such as decreasing drug dose, toxicity, and cost, as well as increasing therapeutic effects. Thus, combination drugs targeting RBD and FP or SH may effectively enhance broad-spectrum antiviral activity against pan-sarbecoviruses, or pan-betacoronaviruses, or even pan-coronaviruses.

## Peptidic and proteinaceous fusion inhibitors interfere with HR1-HR2 bundle assembly

HR2 binds to the exposed HR1 groove to form 6-HB, which is a critical step in mediating virus entry into host cells. Consequently, the HR1-HR2 interface is a prospective target for developing broad-spectrum fusion inhibitors.

### HR2-based peptides preventing the HR1-HR2 interaction

Theoretically, inhibitor peptides that are highly similar or identical to HR2 can bind to the HR1 groove in the intermediate state of S and effectively impede the HR1-HR2 interaction, thereby blocking membrane fusion and virus entry (Fig. 3d). Here, we summarize and discuss HR2-based peptides that interfere with HR1-HR2 bundle assembly (Table 3).

**Table 3:** HR2-based peptides impeding the HR1-HR2 interaction

Name	Sequence		IC50s of viral S-mediated cell-cell fusion (nM)	IC50s of pseudotyped coronaviruses (nM)	IC50s of live coronaviruses (nM)	Protective efficacy	Stage	Refs.
2019-nCoV-HR2P	D_1168_ISGINASVVNIQKEIDRLNEVAKNLNESLIDLQEL_1203_		WT: 180 *VS* EK1:190	WT: 980 *VS* EK1: 2380	N.A.	N.A.		^72^
OC43-HR2P	SLDYINVTFLDLQVEMNRLQEAIKVLNQSYINLKDI		SARS-CoV: 540, MERS-CoV: 390, HCoV-OC43: 660, HCoV-229E: 840, HCoV-NL63: 940	WT > 1270	HCoV-OC43: 930	N.A.		^75^
HY3000	P3	I_1169_SGINASVVNIQKEIDRLNEVAKNLNESLIDLQEL_1203_	P3 SARS-CoV-2: 720	IC50s of WT, Alpha, Beta, Gamma, Kappa, and Delta. P3: 780, 890, 960, 450, 1250, and 1390, respectively. P3-1: 370, 540, 360, 110, 190, and 190, respectively. P3-2: 340, 800, 230, 140, 600, and 270, respectively. P3-3: 230, 370, 100, 110, 430, and 320, respectively. P3-4: 240, 590, 200, 220, 480, and 550, respectively. P4-5: 1590, 1430, 1240, 630, 1060, and 1820, respectively.	WT: 580	N.A.	Clinical trials	^81^ (PN: CN 114437184 B)
	P3-1	ISGINASVVNIQKEIDRLNEVAKNLNESLIDLKEL						
	P3-2	VDLGDISGINASVVNIQKEIDRLNEVAKNLNESLIDLQEL						
	P3-3	VKFGDISGINASVVNIKEEIDRLYEVVKNLNESLIDLQEL						
	P3-4	ISGINASVVNIKEEIDRLNEVAKNLNESLIDLQEL						
	P3-5	ISGINASVVNIQKEIDRLNEVAKELNESLIDLQEL						
EK1	SLDQINVTFLDLEYEMKKLEEAIKKLEESYIDLKEL		WT: 315.0, B.1.1.529: 95, SARS-CoV: 409.3, MERS-CoV: 239.5, HCoV-OC43: 787.6, HCoV-229E: 207.4, HCoV-NL63: 751.0	WT: 1270, Alpha: 1240, Gamma (B.1.1.248): 1250, Omicron: 309, SARS-CoV: 3237, MERS-CoV: 631.8, HCoV-OC43: 1398, HCoV-229E: 3963, HCoV-NL63: 7666, WIV1: 5425, Rs3367: 6014	WT: 2468, Omicron: 1138, MERS-CoV: 802.1, HCoV-OC43: 1554, HCoV-229E: 4375, HCoV-NL63: 3693	Intranasal application of EK1 can prevent and treat infection in mice challenged with HCoV-OC43, MERS-CoV, and SARS-CoV-2	Clinical trials	^75–78^
EK1C4	EK1-GSGSG-PEG4-Chol		WT: 1.3, Omicron: 0.9, SARS-CoV: 4.3, MERS-CoV: 2.5, HCoV-OC43: 7.7, HCoV-229E: 5.2, HCoV-NL63: 21.4	WT: 5.7, Alpha: 5.5, Gamma (B.1.1.248): 6.5, Omicron: 8.6, SARS-CoV: 11.7, MERS-CoV: 11.1, HCoV-OC43: 37.7, HCoV-229E: 12.4, HCoV-NL63: 76.6, WIV1: 30.8, Rs3367: 66.9	WT: 36.6, Omicron: 85.4, MERS-CoV: 4.2, OC43: 24.8, HCoV-229E: 101.5, HCoV-NL63: 187.6	Intranasal application of EK1 can prevent and treat infection in mice challenged with HCoV-OC43 and SARS-CoV-2		^76–78^
EKL1C	EK1-GSG-Chol		WT: 8.0, B.1.1.529: 5.5	WT: 37.0, Alpha: 12.0, Gamma (P1): 46.0, B.1.1.529: 26.1, SARS-CoV: 76.0, MERS-CoV: 48.0, HCoV-OC43: 668, HCoV-NL63: 35.0, WIVI: 218.0, Rs3367: 46.0	WT: 3.0, Omicron: 182.2, HCoV-OC43: 281	Intranasal application of EKL1C can prevent and treat infection in mice challenged with SARS-CoV-2 and HCoV-OC43		^77,89^
EK1C16	EK1-GSGSG-PEG4-C16		MERS-CoV: 10.0, HCoV-OC43: 10.0	WT: 480, Alpha: 190, Beta: 430, Gamma (B.1.1.248): 260, Delta: 110, Omicron: 230, MERS-CoV: 100, SARS-CoV: 170, WIV1: 150, Rs3367: 300	HCoV-OC43: 70.0, Omicron: 750	N.A.		^93^
EK1P4HC	EK1-GSGSG-PEG4-25-HC		N.A.	WT: 800, Alpha: 2280, Beta: 620, Gamma (B.1.1.248): 480, Delta: 110, SARS-CoV: 350, MERS-CoV: 100	HCoV-OC43: 410, HCoV-229E: 480	Preventing and treating infection in mice challenged with HCoV-OC43		^94^
IPB02	ISGINASVVNIQKEIDRLNEVAKNLNESLIDLQELK (Chol)		WT: 31.6, D614G: 27.7	WT: 148.8, D614G: 88.1, SARS-CoV: 304, MER-CoV: 642, HCoV-NL64: 568.9, HCoV-229E: 604.8	N.A.	N.A.		^86^
IPB02V1	ISGINASVVNIQKEIDRLNEVAKNLNESLIDLQEL-PEG8-K (Chol)		WT: 1.1, D614G: 0.6	WT: 14.3, D614G: 21.5, SARS-CoV: 33.6, MER-CoV: 66.9, HCoV-NL64: 62.5, HCoV-229E: 203.9				
IPB02V2	DISGINASVVNIQKEIDRLNEVAKNLNESLIDLQEL-PEG8-K (Chol)		WT: 0.6, D614G: 0.4	WT: 18.1, D614G: 20.8, SARS-CoV: 56.5, MER-CoV: 55.3, HCoV-NL64: 67.5, HCoV-229E: 535.7				
IPB02V3	EISGINASVVNIQKEIDRLNEVAKNLNESLIDLQEL-PEG8-K (Chol)		WT: 0.4, D614G: 0.2	WT: 17.5, D614G: 14.4, SARS-CoV: 48, MER-CoV: 38.5, HCoV-NL64: 75.8, HCoV-229E: 545.7				
IPB02V4	ELSGINASVVNLQKEIDRLNEVAKNLNESLIDLQEL-PEG8-K (Chol)		WT: 0.3, D614G: 0.1	WT: 15.2, D614G: 26.3, SARS-CoV: 63, MER-CoV: 22.4, HCoV-NL64: 66.3, HCoV-229E: 502				
IPB02V5	SLTQINASVVNLQKEIDRLNEVAKNLNESLIDLQEL-PEG8-K (Chol)		WT: 2.1, D614G: 1.3	WT: 23.5, D614G: 27.5, SARS-CoV: 65.9, MER-CoV: 68.4, HCoV-NL64: 70.8, HCoV-229E: 1128.4				
MERS-LP	SLTQINTTLLDLTYEMLSLQQVVKALNESYIDLKEL-PEG8-K (Chol)		WT: 102.9, D614G: 79.1	MERS-CoV: 82.9; WT, D614G, SARS-CoV, HCoV-NL64, and HCoV-229E: more than 2500				
OC43-LP	SLDYINVTFLDLQDEMNRLQEAIKVLNQSYINLKDI-PEG8-K (Chol)		WT: 4.1, D614G: 2.4	WT: 82.8, D614G: 97.5, SARS-CoV: 250.4, MER-CoV: 5.2, HCoV-NL64: 416.5, HCoV-229E: 2008.8				
EK1V1	SLDQINVTFLDLEYEMKKLEEAIKKLEESYIDLKELLK (Chol)		WT: 273.5, D614G: 214.5	WT: 2672, D614G: 1790, SARS-CoV: 4370, MER-CoV: 500, HCoV-NL64: 487, HCoV-229E: 1255.6				
EK1V2	SLDQINVTFLDLEYEMKKLEEAIKKLEESYIDLKELL--PEG8-K (Chol)		WT: 0.9, D614G: 0.5	WT: 87.2, D614G: 106.5, SARS-CoV: 252, MER-CoV: 1.1, HCoV-NL64: 59.4, HCoV-229E: 503.3				
longHR2_42	P_1162_DVDLGDISGINASVVNIQKEIDRLNEVAKNLNESLIDLQ_1201_		WT: 1.3	WT: 1.1, Delta: 0.9, Omicron: 4.1	WT: 1.5, Alpha: 0.6, Delta: 5; Omicron: 15.6	N.A.		^80^
longHR2_45	K_1157_NHTSPDVDLGDISGINASVVNIQKEIDRLNEVAKNLNESLIDLQ_1201_		WT: 1.6	N.A.	N.A.	N.A.		^80^
[SARS_HRC_-PEG_4_]_2_-chol	HRC-PEG_4_-chol-PEG_4_-HRC The sequence of HRC: D_1168_ISGINASVVNIQKEIDRLNEVAKNLNESLIDLQEL_1203_		For WT, D614G, Alpha, Beta, SARS-CoV: 3~10	WT: ~300 (VeroE6 cells), ~5 (VeroE6-TMPRSS2 cells)		It can prevent SARS-CoV-2 transmission in ferrets by intranasal application		^87^

*WT,* wild type (of SARS-CoV-2). *N.D*., indicates below neutralizing threshold. *N.A*., not application. *PN,* patent number. *CN*, China

#### HR2P and unmodified-EK1 as broad-spectrum anti-coronaviral fusion inhibitors

The first reported fusion inhibitory peptide against HIV-1 infection was SJ-2176, designed based on the HR2 segment of HIV-1 gp41.^69^ Subsequently, many peptide-based anti-coronaviral fusion inhibitors derived from HR2 domain have been reported, including the anti-SARS-CoV peptide (CP-1), anti-MERS-CoV peptide (MERS-HR2P), and anti-SARS-CoV-2 peptide (2019-nCoV-HR2P).^70–73^ However, these HR2-based peptides exhibit weak cross-inhibitory activities against heterologous HCoVs, possibly due to the different length of HR1 sequences between α-CoV and β-CoV.^74^ Following the screening of fusion inhibitor peptides based on HR1 or HR2 derived from five HCoVs (SARS-CoV, MERS-CoV, HCoV-OC43, HCoV-229E, and HCoV-NL63), OC43-HR2P was selected due to its broad neutralizing activity (sub-micromolar) against the tested HCoVs.^75^ In addition, EK1—the OC43-HR2P peptide modified by E-K mutation to enhance solubility and stability—exhibits an enhanced inhibitory activity against the five HCoVs and certain SL-CoVs. Furthermore, intranasal application of EK1, before or after viral challenge, prevents HCoV-OC43 and MERS-CoV infections in mice.^75^ In addition, EK1 effectively inhibits SARS-CoV-2 S-mediated membrane fusion and pseudovirus infection with IC50s of 0.31 μM and 2.37 μM, respectively, showing the comparable inhibitory activity to 2019-nCoV-HR2P.^72,76^ Moreover, EK1 can effectively neutralize the Omicron pseudovirus infection with a sub-micromolar IC50.^77,78^ Nasal administration of EK1 also prevents and treats SARS-CoV-2 infection in mice; it also exhibits good safety profiles in different animal models, even at high dosages.^76,78^ The complex structure of 6-HB formed by SARS-CoV-2 HR1 and EK1 indicates that EK1 fits well into the groove formed by two adjacent HR1s, thus probably blocking viral 6-HB formation and subsequent preventing membrane fusion, further supporting the key role of the HR1 groove as a viable antiviral target.^78^ Moreover, EK1 can adapt well to the HR1 groove of different HCoVs and exhibit high affinity to these HR1s, thus exerting broad inhibition activity against various coronaviruses.^78^

Yan et al. reported that EK1 with dual Q1004E/N1006I mutations has an improved ability to bind to the HR1 groove, probably enabling an enhanced efficacy against the COVID-19 infection than primary EK1.^79^ Moreover, Yang et al. designed two HR2 peptides with 6 or 11 extended residues in the N-terminal region of HR2 (longHR2_42 or longHR2-45, respectively) that were 100-fold more potent than the reported short, unmodified HR2 peptide against SARS-CoV-2 infection.^80^ The extended HR2 peptide (longHR2_42) inhibits pseudovirus infection of SARS-CoV-2 Omicron and other VOCs with almost nanomolar IC50s.^80^ Thus, the N-terminal region of HR2 (or the C-terminal region of SH) may offer new possibilities for the design of more potent fusion inhibitors against SARS-CoV-2.^80^ Overall, the optimal length and sequence for HR2-based fusion inhibitors warrant further investigation.

Importantly, Wang et al. reported that P3—the SARS-CoV-2 HR2 (I1167-L1203) peptide—shows strong inhibitory activity against authentic SARS-CoV-2 and its PsV with IC50s of 0.32 μM and 0.58 μM, respectively. In addition, P3 can prevent SARS-CoV-2 S-mediated cell-cell fusion.^81^ Importantly, HY3000, the P3-modified peptides, exhibit more potent cross-inhibitory activity against pseudovirus infection with SARS-CoV-2 and its variants (e.g., Alpha, Beta, Gamma, Kappa, Delta, and Omicron) (CN 114437184 B). Indeed, the efficacy of inhalable formulations of HY3000 and EK1 against SARS-CoV-2 infection is currently being assessed in clinical trials in China, which may provide new avenues for the prevention or treatment of COVID-19 and the potential emergence of novel coronaviruses or other zoonotic coronaviruses.

#### HR2- and EK1-based lipopeptides as broad-spectrum anti-coronaviral fusion inhibitors

The viral inhibitory activity of EK1 or HR2P against SARS-CoV-2 was measured at micromolar concentrations and was modest relative to the HIV-1 gp41 HR2 peptide, which prevents HIV infection at nanomolar concentrations.^82,83^ Given that lipid-labelled peptides have an extended half-life and can localize at high concentrations more readily where S-directed membrane fusion occurs in the endosome or on the cell surface, they can provide broader and more potent antiviral effects.^84^ This was confirmed by an almost 1000-fold increase in the anti-MERS-CoV effect using a lipid-modified peptide named MERS-CoV-HR2P-M2.^85^ Hence, Jiang et al. designed EK1C4—EK1 modified with a polyethylene glycol-conjugated (PEGylated) lipopeptide—that inhibits SARS-CoV-2 S-mediated membrane fusion, pseudovirus infection, and live virus infections with IC50s of 1.3, 5.7, and 36.6 nM, which are about 240-, 150-, and 67-fold more potent than the original EK1 peptide, respectively.^76^ In addition, EK1C4 exhibits enhanced cross-inhibitory activity against pseudovirus infection of the five HCoVs and three zoonotic coronaviruses (WIV1, Rs3367, and SHC014).^76^ EK1C4 also effectively inhibits infection caused by SARS-CoV-2 variants, including Alpha, Gamma, and Omicron, with nanomolar IC50s in vitro.^77,78^ Moreover, nasal administration of EK1C4 protects against SARS-CoV-2 and HCoV-OC43 infections with satisfactory safety profiles in vivo.^76,78^

Zhu et al. also reported IPB02—the original HR2 peptide modified with a C-terminal cholesterol—that inhibits SARS-CoV-2 S-mediated membrane fusion and pseudovirus infection, with IC50s of 31 nM and 148 nM, respectively.^86^ In addition, IPB02 derivatives—the modified IPB02 peptides with a PEG8-linked cholesterol—exhibit potent cross-inhibitory activity against the five HCoVs.^86^ Importantly, EK1V2 which was modified similar to IPB02 derivatives exhibits an enhanced antiviral potency than EK1 or EK1C4.^86^

Notably, the dimeric HR2-modified lipopeptide, named [SARS_HRC_-PEG_4_]_2_-chol, that modified with two original HR2 peptides linked by PEG_4_-cholesterol-PEG_4_, shows an enhanced cross-neutralizing activity (nanomolar) by blocking 6-HB formation and preventing virus-host membrane fusion.^87^ In addition, daily intranasal administration of [SARS_HRC_-PEG_4_]_2_-chol provides complete protection for ferrets against SARS-CoV-2 infection in vivo through direct-contact transmission.^87^ This indicates that HR2-based poly-peptides/lipopeptides may serve as a promising intranasal prophylaxis to prevent SARS-CoV-2 transmission in vivo.^87^

Considering that PEG linkers might affect peptide stability or elicit anti-PEG antibodies in vivo, EKL1C—EK1 with a de-PEGylated lipopeptide—was designed.^88,89^ Although EKL1C shows enhanced antiviral activity compared to EK1 against SARS-CoV-2, its VOCs, other HCoVs, and zoonotic coronaviruses, its entry-inhibition activity is still weaker than that of EK1C4.^77,78,89^ Moreover, EKL1C protects against SARS-CoV-2 and HCoV-OC43 infections as a prophylactic or therapeutic agent in mice.^89^ Importantly, EKL1C is more resistant to proteolytic enzymes and has a higher thermostability than EK1C4.^89^ Although, cholesterol-modified peptides have not been approved for clinical applications, a palmitic acid-based HIV lipopeptide vaccine was shown to be safe and effective in phase 2 clinical trials.^90^ Another study reported that 25-hydroxycholesterol (HC), the ideal safety profile as a natural cholesterol metabolite in humans, shown potent antiviral potency against HCoV-OC43 infection in vivo and in vitro.^91,92^ Accordingly, Jiang et al. synthesized a palmitic acid-based lipopeptide (EK1-C16) and 25-hydroxycholesterol-modified peptide (EK1P4HC) that are effective on preventing infection caused by SARS-CoV-2 VOCs, as well as the tested HCoVs and zoonotic CoVs.^93,94^

Taken together, HR2- and EK1-derived lipopeptides are promising candidates as broad-spectrum anti-coronaviral fusion inhibitors.

### Proteinaceous inhibitors blocking the HR1-HR2 bundle assembly

Fusion inhibitors based on the HR1 domain can theoretically block virus infection by binding to HR2, and, therefore, preventing viral 6-HB formation (Fig. 3e). However, HR1-based peptides, including SARS-CoV-HR1P, MERS-HR1P, and 2019-nCoV-HR1P, all fail to exert an effective neutralizing activity.^71,72,75,95^ This may be due to their strong propensity to aggregate. Nevertheless, Dang et al. designed HR1MFd—SARS-CoV-2 HR1 (E918-V952) linked with the folded motif to form an HR1 trimer protein—that showed moderate cross-neutralizing activity against SARS-CoV-2, its VOCs (Alpha and Omicron), SARS-CoV, and MERS-CoV, albeit at micromolar IC50s (Table 4).^96^

**Table 4:** Proteinaceous inhibitors blocking the HR1-HR2 interaction

Name	Constructs	IC50s of viral S-mediated cell-cell fusion (nM)	IC50s of pseudotyped coronaviruses (nM)	IC50s of Live coronaviruses (nM)	Protective efficacy	Refs.
HR1MFd	HR1(E918-V952)-Fd	WT: 740	WT: 1230, Alpha: 2350, Omicron: 1060, SARS-CoV: 2130, MERS-CoV: 2860	N.A.	N.A.	^96^
5-HB-H2	HR1-HR2-HR1-HR2-HR1-His (HR1:E918-L966; HR2: D1168-L1203)	SARS-CoV-2: 630	WT: 590, Beta: 3080, Omicron: 1630, Kappa: 3250, Delta: 2660, RaTG13: 2510, PANG/GD: 1.0, SARS-CoV: 1670, WIV1: 2460, HKU3: 1.0	N.A.	N.A.	^49^
SARS-CoV-2-5-Helix	HR1-HR2-HR1-HR2-HR1-His (HR1:L922-Q965; HR2: D1165-G1204)	SARS-CoV-2: 127	WT: 243, Alpha: 363.6, Beta: 169.7, Gamma (P.1): 141.8, Delta: 368.9, Omicron: 140.7, lambda: 238.2	SARS-CoV-2: 293, Omicron: 279	N.A.	^95^
GL25E	GRFT-(G4S)5-EK1	SARS-CoV-2: 4.4, SARS-CoV: 30.1, MERS-CoV: 4.9, HCoV-NL63: 0.40, HCoV-229E: 0.33, HCoV-OC43: 0.27	WT: 8.7, SARS-CoV: 62.1, MERS-CoV: 0.2, HCoV-NL63: 0.5, HCoV-229E: 1.8, HCoV-OC43: 0.15	SARS-CoV-2: 29.8, HCoV-OC43: 0.2, HCoV-229E: 177.2	Intranasal application of GL25E can prevent and treat infection in mice challenged with HCoV-OC43	^101^
FL-EK1	FN3-Linker-EK1	SARS-CoV-2: 68.5 HCoV-OC43: 398.2	WT: 90.6, Alpha: 114.5, Beta: 201.2, Gamma (P.1): 373.1, Delta: 133.0, Omicron: 297.5, Lambda: 88.5, Eta: 179.2, Kappa: 230.9, HCoV-OC43: 1593	SARS-CoV-2: 25.3, Delta: 281.2, HCoV-OC43: 207	Intranasal application of FL-EK1 can prevent and treat infection in mice challenged with HCoV-OC43 and SARS-CoV-2 Delta variant	^102^

*WT,* wild type (of SARS-CoV-2). *N.D*., indicates below neutralizing threshold. *N.A*., not application. Fd, T4 foldon

To improve stability and solubility, small recombinant proteins, denoted 5-HB, comprising HR1 and HR2, with one HR1 groove remaining unoccupied and capable of binding to one HR2, have been successfully designed for HIV, MERS-CoV, and SARS-CoV-2.^49,83,95,97^ The SARS-CoV-2 5-HB protein showed broad neutralizing activity against pseudovirus infections of SARS-CoV-2 and its variants, as well as certain zoonotic coronaviruses, with picomolar to micromolar IC50s (Table 4).^49^ Although the entry-inhibition efficacy of 5-HB is slightly weaker than that of HR2 or EK1, 5-HB can bind to the pre-exposed HR2 domain in pre-S state, whereas HR2 or EK1 only interact with the HR1 groove in the intermediate state of S, not the pre- or post-fusion states of S (Fig. 3d, e).^49^ Thus, as membrane fusion inhibitors, 5-HB protein may be a better preventative candidate than HR2-based peptides.^49^ Mechanistically, 5-HB protein provides an extended hydrophobic groove to accommodate HR2, thereby preventing viral 6-HB formation, thus impeding membrane fusion. Notably, 5-HB binds to HR2 at serological and endosomal pH with a similar binding affinity, probably indicating its entry-inhibition capacity may not be affected when SARS-CoV-2 enters via a membrane fusion pathway or an endosomal route.^49^ In addition, Liu et al. also reported a similar SARS-CoV-2-5-Helix protein that prevents infection with authentic SARS-CoV-2 and the Delta variant, with sub-micromolar IC50s (Table 4).^95^ Collectively, these studies suggest that 5-HB protein may serve as a promising broad-spectrum antiviral fusion inhibitor.^49,95^

Lectin griffithsin (GRFT), comprising 121 amino acids, is an anti-HIV microbicide that is currently in clinical trials for HIV prevention. In addition, GRFT exhibits a high affinity for mannose recognition and broad-spectrum antiviral activity.^98^ Meanwhile, GRFT can prevent SARS-CoV and SARS-CoV-2 infections by interacting with glycosylation sites in the S1 subunit, potentially the RBD region.^99,100^ Cai et al. showed that GRFT, combined with EK1, exerts excellent synergistic inhibitory activity against SARS-CoV-2 infection.^99^ Moreover, a recombinant protein, GRFT-L25-EK1 (GL25E), exhibits more potent neutralizing activity against SARS-CoV-2 and its VOCs, as well as the five HCoVs, compared with GRFT or EK1 alone (Table 4).^101^ In addition, the cost of manufacturing GL25E with an *E.coli* expression system at a large scale is more economical than the synthesis of cholesterol-conjugated EK1 lipopeptides, such as EK1C4, EKL1C, and EK1-C6. As such, GL25E has better prospects for developing clinical drugs to prevent and treat COVID-19 and other coronaviruses.^101^ To increase the half-life of EK1 in vivo, Duan et al. engineered a recombinant protein, named FL-EK1, comprising a modified fibronectin type domain (FN3) with reversible albumin-binding ability linked to EK1.^102^ Similar to EK1, FL-EK1 exhibits cross-neutralizing activity against SARS-CoV-2, the tested VOCs, as well as HCoV-OC43 in vitro, albeit with slightly enhanced inhibitory activity compared to EK1 alone (Table 4).^102^ Moreover, FL-EK1 protects mice from the Delta variant and HCoV-OC43 challenge in vivo. Notably, the half-life of FL‐EK1 was 15‐fold longer than that of EK1 in a mouse model, suggesting that it has a better pharmacokinetic profile and is a promising long-acting antivirals.^102^ Overall, owing to the longer half-life and lower cost of large-scale production of proteinaceous drugs, compared with peptidic drugs, strategies to enhance the pharmacokinetic properties of anti-CoV proteinaceous inhibitors, such as IgG Fc-binding peptide conjugation and IgG Fc-bivalent proteins, warrant further investigation.

Overall, these fusion inhibitors (e.g., nAbs, 5-HB proteins, and HR2- or EK1-based peptides/lipopeptides) could exhibit different antiviral mechanisms by interacting with the targetable S2 fusion elements (the FP, SH, and HR1-HR2 bundle) in distinct conformations of S protein (Fig. 3). Anti-FP nAbs can bind to the uncovered S2ʹ site and FP in the intermediate S protein, inhibiting S2ʹ cleavage and/or preventing FP insertion into the host cell membrane. Anti-SH nAbs are expected to bind to SH in pre-S, probably disrupting the SH three-helical bundles and sterically block SARS-CoV-2 S fusogenic rearrangements. HR2/EK1-based peptides bind to the HR1 groove in the intermediate state of S and 5-HB protein bind to HR2 in pre-S trimer. All would impede 6-HB formation to prevent membrane fusion. During the endocytosis of viral particles, the entry-inhibition activity of anti-FP and anti-HR1 inhibitors may be influenced due to the relatively low concentration within the endosome. However, HR2- and EK1-derived lipopeptides may have remained active during the endocytosis of virions, since they can be more readily localized at high concentrations where S-directed membrane fusion occurs, even in the endosome. Moreover, SH- and HR2-targeted inhibitors can pre-bind virions floating outside of the plasma membrane, and thus could exhibit an effective neutralizing activity during the endocytosis of viral particles, thereby providing a longer binding window for preventive therapy relative to anti-FP and anti-HR1 inhibitors.

## Small-molecule compounds that block the HR1-HR2 interaction

Compared with bio-macromolecule drugs, most small-molecule compounds have the advantage of lower cost, higher oral bioavailability, higher adherence, higher stability and more ease of transportation and storage, making them a promising candidate for clinical applications. Currently, many broad-spectrum compounds (e.g., Remdesivir, Molnupiravir, VV116, and Azvudine) have already been approved for the treatment of COVID-19 by targeting SARS-CoV-2 RNA-dependent RNA polymerase (RdRp).^103–105^ In addition, several candidate compounds are undergoing clinical trials that could enrich anti-COVID-19 drugs.^105^ Moreover, small-molecule compounds reportedly protect against SARS-CoV-2 infection by blocking the HR1-HR2 bundle assembly, paving a potential way for screening and designing broad-spectrum antiviral inhibitors.

Pu et al. synthesized a series of small-molecule compounds FD001 to FD0012. FD001, for instance, exhibits broad-spectrum antiviral activity against pseudovirus infection mediated by SARS-CoV-2 and its VOCs as well as MERS-CoV and SARS-CoV, with sub-micromolar IC50s.^106^ In addition, FD001 and its analogues effectively inhibit infection caused by authentic SARS-CoV-2 and Delta, with IC50s of 13 ~ 410 nM, which are similar (Delta) or superior (SARS-CoV-2) to remdesivir.^106^ Mechanistically, FD001 and its analogues are proposed to interfere with the HR1-HR2 bundle formation and/or to target the virus membrane to inactivate cell-free virions.^106^

Salvianolic acid (Sal-C)—a hydrophilic compound isolated from the traditional Chinese medicine, Danshen—exhibits potent antiviral activity against SARS-CoV-2 pseudovirus and authentic SARS-CoV-2 infections with IC50s of 3.85 μM and 3.41 μM, respectively.^107^ In addition, Sal-C may impede the 6-HB fusion core assembly by interacting with residues S940, T941, A942, L945, K947, L948, and Q949 in the conserved hydrophobic pocket of the SARS-CoV-2 HR1 groove.^107^ Moreover, Davide Gentile et al. also confirmed the efficacy of Sal-C in preventing SARS-CoV-2 infection. They demonstrated the importance of the interaction between Sal-C and residues (K933 and K947) for occupancy of the hydrophobic pocket.^108^

Umifenovir (Arbidol)—a broad-spectrum anti-influenza drug—has been used clinically in Russia and China with good efficacy and safety. It blocks influenza virus entry into host cells by disrupting virus‐mediated membrane fusion.^109^ More specifically, Arbidol binds to the hydrophobic cavity of the HA trimer and inhibits pH-induced post-fusion conformational transition related to membrane fusion.^110^ Recently, studies have reported that Arbidol is useful for treating SARS-CoV, MERS-CoV, and SARS-CoV-2 infections.^111,112^ Molecular dynamics and structural analysis revealed that Arbidol interacts with residues in the central CH-HR1 helix via hydrogen bonding and van der Waals forces.^113^ Thus, Arbidol could effectively interfere with the trimerization of the SARS-CoV-2 S protein, which is critical for virus entry.^113^ Another study reported that Arbidol effectively prevents SARS-CoV-2 infection with an IC50 value of 4.11 μM by blocking viral attachment and releasement from endolysosomes.^114^ Furthermore, a retrospective cohort study reported that post-exposure prophylactic application of Arbidol is related to a reduced risk of COVID-19 infection in hospital and community settings.^115^ That is, a clinical trial reported that Arbidol markedly accelerates the negative conversion rate of SARS-CoV-2 and improves the recovery of COVID-19 patients.^116^ Moreover, Arbidol combined with lopinavir/ritonavir improves the negative conversion rate for coronaviruses and chest computed tomography scans compared with lopinavir/ritonavir alone.^117^ However, a retrospective study suggested that additional Arbidol does not enhance prognosis, or facilitate viral clearance, in non-Intensive Care Unit patients.^118^ Therefore, understanding the potential drug targets and action-mechanisms of Arbidol may guide the future design of new antivirals.

Moreover, certain Food and Drug Administration-approved drugs reportedly help combat SARS-CoV-2 infection. For example, Posaconazole, an antifungal drug, can inhibit SARS-CoV-2 and its variant infections. It can bind to the conserved *“*E1182-L1186L-L1193*”* motif located in HR2 with high affinity, thereby interfering the 6-HB fusion core formation and then blocking membrane fusion.^119^ In this way, Posaconazole can serve as a broad-spectrum fusion inhibitor against the ongoing SARS-CoV-2 variants.^119^ In addition, itraconazole and estradiol benzoate could interact with the HR1 of several HCoVs, including SARS-CoV-2, SARS-CoV, and MERS-CoV, and exhibit broad neutralizing activity in vitro.^120^ Meanwhile, biflavone-based anti-HIV drugs, such as hinokiflavone and robustaflavone, could prevent SARS-CoV-2 S-mediated virus entry by blocking the HR1-HR2 interaction.^121^

Recently, a fluorescent polarization detection method based on 5-HB protein and HR2 that mimics 6-HB formation during virus-host membrane fusion was used to evaluate and screen small-molecule fusion inhibitors against SARS-CoV-2.^122^ However, attempts to identify anti-SARS-CoV-2 fusion inhibitors have been unsuccessful, largely due to the small size of compounds library used. Although this approach has identified small-molecule compounds targeting the HIV fusion core, their effects on neutralizing virus infection are relatively moderate, with IC50s of 18 ~ 38 μΜ.^123^ Future attempts for isolation of small-molecule inhibitors targeting SARS-CoV-2 fusion core might, therefore, require optimized screening systems and/or expand small-molecule libraries.

Taken together, these studies suggest that small-molecule compounds that block 6-HB formation have the potential to treat or prevent the emerging COVID-19 infection. Moreover, the screening of antiviral small-molecule inhibitors based on 6-HB fusion core structure may be ideal and feasible for novel coronaviruses.

## Vaccines based on the SARS-CoV-2 S2 fusion subunit

The emergence and rapid dominance of Omicron sub-lineages (BA.2, BA.4/5, BA.2.12.1, XBB, and BQ.1) show striking immune evasion against most antibodies and vaccines.^124–126^ This has required rapid updating of vaccine S antigen strategies to adapt to the ongoing antigenic drift pressure. Notably, the targetable S2 subunit has been shown to harbor promising cross-reactive antibody epitopes for the design of broad-spectrum vaccines.^58,127,128^ Recently, a membrane-bound SARS-CoV-2 S2 (686-1211) DNA vaccine was shown to induce cross-reactive nAbs in mice that neutralize various animal and human α‐ and β‐CoVs (including the Omicron subvariants) in vitro and to provide certain degree of protection against SARS-CoV-2 challenge in vivo.^129^ Moreover, another study has also reported that a multivalent S2 nanoparticle-based vaccine can induce cross-reactive antibodies and provide effective protection against SARS-CoV-2 variants and PANG/GD challenges in vivo.^130^

### Vaccines design based on the conserved fusion peptide epitope

Universal vaccine candidates against highly variable pathogenic viruses, including HIV-1 and influenza, have been guided based on the conserved FP region recognized by a broad-spectrum nAb.^131,132^ It follows that cross-reactive vaccines containing the conserved FP epitope across the coronavirus subfamily can induce the generation of FP-specific nAbs that might help to resist viral escape.^133,134^ In addition, FP vaccines based on SARS-CoV-2 and porcine epidemic diarrhea virus (PEDV, a α-CoV subgenus belonging to Pedacovirus) have been shown to provide similar clinical protection against PEDV challenge in a porcine model.^135^ Moreover, receptor-induced FP exposure may be blocked by prefusion-stabilizing 2P mutations, indicating that current mRNA vaccines may require further optimization to induce antibody responses against FP.^136^ Therefore, immunogens that reveal the FP epitope may elicit more potent cross-reactive nAbs and can be used as probes for vaccine design. Notably, cross-reactive CD4^+^ T cell immunity to SARS-CoV-2 may be provoked by highly conserved FP, suggesting that these cells may provide intramolecular assistance for FP-targeted antibody production.^137^

Overall, these findings indicate that the targetable FP epitope may guide the design of pan-coronavirus vaccines. Thus, further understanding of the dynamic change of FP structure and the mechanism of interaction between FP and host cell membrane is critical for making clear the key process involved in virus entry, which may promote the development of structure-based FP immunogen design and the screening of FP-based antivirals.

### Vaccines design based on the conserved HR bundle

The HR1-HR2 bundle is also an alternative targetable S2 element for vaccine design. For example, Ma et al.^138^ developed covalently conjugated nanoparticle vaccines displaying either SARS‐CoV‐2 RBD or RBD-HR (HR1–HR2) on the surface.^139^ Notably, the RBD-HR chimeric nanoparticle vaccine could elicit higher titers of HR-specific nAbs, providing cross-protection against diverse human α- and β-CoV PsVs relative to the RBD nanoparticle vaccine.^139^ In particular, the RBD-HR nanoparticle vaccine induces more potent cross-reactive T-cell and B-cell immune responses against variable CoVs, laying a foundation for developing universal CoV vaccines.^139^ Notably, their results suggest that the widespread neutralization of nanoparticle vaccine may be modulated by S2-based antibodies.^139^ Another study also reported a novel subunit vaccine, RBD-HR/trimer, that directly links SARS-CoV-2 RBD and HR (HR1-HR2) in a tandem manner and automatically assembles into a trimeric recombinant RBD/HR protein. The RBD-HR/trimer vaccine produced potent nAb responses against SARS-CoV-2 and the tested VOCs in vitro and provided protection against Omicron and Delta challenges in vivo.^140^

More recently, Pang et al. designed a variant-proof SARS-CoV-2 S2 subunit vaccine, HR121, that targets the more conserved HR1 domain of the S2 subunit.^141^ The recombinant HR121 antigen comprises HR1–HR2–HR1 and forms a stable asymmetric 6-HB conformation via the HR121 dimer, with two surface grooves formed by two adjacent HR1s remaining unoccupied, thereby providing two possible binding sites for exogenous HR2 in the intermediate state of S.^141^ Importantly, high titers of cross-reactive nAbs were induced in both HR121-immunized rabbits and rhesus monkeys. This prevented infection with SARS-CoV-2 and the Omicron BA.2 variant in vitro and in vivo.^141^ Notably, HR121 vaccination produced potent nAb responses in human ACE2 transgenic mice, Syrian golden hamsters, and rhesus monkeys, providing nearly complete protection against ancestral SARS-CoV-2 infection, as well as effective protection against the Omicron BA.2 variant infection in Syrian golden hamsters.^141^ Notably, HR121, which possesses the HR1 groove, is a better vaccine antigen than HR12 that designed with the intact 6-HB scaffold. Although the binding Ab titers elicited by the HR121 antigen were nearly 7.5-fold lower than those elicited by the HR12 antigen, they were more potent in neutralizing SARS-CoV-2 pseudovirus infection in immunized rabbits, with 34.4-fold higher than those elicited by HR12, indicating that Abs targeting the HR1 groove may be more effective against SARS-CoV-2 infection.^141^

Collectively, cross-reactive nAbs that recognize the targetable HR bundle, hold promise for the development of HR-based universal vaccines. In particular, HR121 is a promising variant-proof COVID-19 vaccine candidate for providing a novel cryptic epitope to combat pan-coronaviruses.

## SARS-CoV-2 S-specific T cell responses

Virus-specific humoral immunity and cellular immunity exhibited a strong synergistic effect in protecting the host from virus infection.^142,143^ This adaptive immune response was reliant on B cells for producing antibodies, as well as on T cells for alleviating virus-infected cells (CD8^+^ T cells) and inducing helper and effector functions (CD4^+^ T cells), all of which are critical to the success of SARS-CoV-2 vaccines.^142^ Memory T cells combined with antibody responses to form the basis of protective immunity against SARS-CoV-2.^144,145^ In addition, protective nAbs may wane within months after infection or vaccination, whereas coronavirus-induced memory T cells can be maintained for decades and durably persist within humans.^146–148^

SARS-CoV-2-specific CD4^+^ and CD8^+^ T cell responses can be induced following SARS-CoV-2 infection or vaccination.^147,149,150^ These functional T cells play a potential role in reducing COVID-19 disease severity and in providing antiviral protection.^142,151–153^ The early production of functional SARS-CoV-2-specific T cells is related to mild COVID-19 and enhanced viral clearance.^152^ One previous study reported that circulating SARS-CoV-2-specific CD4^+^ and CD8^+^ cells were detected in 100% and 70% of COVID-19 recovered patients, respectively.^154^ The SARS-CoV-2-specific CD4^+^ T cell responses to spike were closely related to the magnitude titers of anti-SARS-CoV-2 S antibodies, because CD4^+^ T cells would contribute to B cell-mediated antibody production.^154^ In addition, SARS-CoV-2 S-specific CD4^+^ T cells can be identified in 40 ~ 60% of SARS-CoV-2-unexposed individuals, indicating cross-reactive T cell recognition between SARS-CoV-2 and human endemic coronaviruses (e.g., 229E, OC43, HKU3, and NL63).^154^ SARS-CoV-2 S-specific CD4^+^ T cells from uninfected individuals primarily react against the S C-terminal epitopes of SARS-CoV-2 and human endemic coronaviruses, suggesting that S2 subunit may contribute to stimulating the production of pre-existing cross-protective CD4^+^ T cells.^155^

COVID-19 vaccines can elicit the generation of SARS-CoV-2 S-specific CD4^+^ and CD8^+^ memory T cells.^148^ A previous study reported that SARS-CoV-2 S-specific memory T cells derived from the ancestral strain exposed to donors or vaccinees could effectively recognize the spike of the variants. Notably, 93% (CD4^+^) and 97% (CD8^+^) of T cell epitopes are highly conserved across these variants, suggesting that mutations in these variants would unlikely disrupt the total SARS-CoV-2 T cell reactivity.^156^ Six months post-vaccination, 84% (CD4^+^) and 85% (CD8^+^) of memory T cell responses induced by SARS-CoV-2 vaccines were preserved against Omicron, whereas Omicron RBD-specific memory B cells were reduced.^157^ The median 11 (CD4^+^) and 10 (CD8^+^) of spike-specific T cell epitopes were recognized by each vaccinated donor, with average preservation being more than 80% for Omicron.^157^ Notably, some highly conserved spike-specific CD4^+^ T cell epitopes were located in the targetable S2 elements, such as K_921_LIANQFNSAIGKIQ_935_ in HR1, S_816_FIEDLLFNKVTLAD_830_ in FP, L_1141_QPELDSFKEELDKY_1155_, D_1146_SFKEELDKYFKNHT_1160_ and T_1136_VYDPLQPELDSFKE_1150_ in SH.^157^ Another study reported dominant T cell epitopes for robust spike-specific responses induced by two vaccine doses. The L_821_LFNKVTLA_829_ (located in FP) and V_976_LNDILSRL_984_ (located in HR1) epitopes of CD8^+^ (HLA-A^*^02:01) T cells from the ancestral strain were 100% and 97.6% conserved, respectively, across the indicated SARS-CoV-2 VOCs.^158^

Overall, functional SARS-CoV-2 S-specific T cells play an important role in the recognition of virus-infected cells and inducing a protective immune response against COVID-19.

## Prospects and conclusions

This current review summarizes the most recently developed broad-spectrum fusion inhibitors and vaccine candidates targeting the conserved fusion elements in SARS-CoV-2 S2 subunit. In particular, we highlight the role of the more targetable S2 elements, including the fusion peptide, HR1-HR2 bundle, and stem helix region, in antibody recognition and antiviral design. Moreover, we provide a detailed summary of the characteristics and action-mechanisms for each class of cross-reactive fusion inhibitors (e.g., nAbs, peptides, proteins, and small-molecular compounds), which have the potential to further enrich the family of antiviral drugs and should guide and promote future design of S2-based vaccines.

Novel S2-based fusion inhibitors also need further study, such as nanobodies and aptamers, that can specifically recognize antigen epitopes with high affinity.^159,160^ Numerous studies have reported that nanobodies and aptamers can effectively neutralize SARS-CoV-2 infection by blocking the RBD-ACE2 interactions.^161–164^ Due to their small size and unique conformation, they are more likely to bind the cryptic epitopes that are usually inaccessible to conventional antibodies. Hence, they have the potential to perform more potent cross-neutralizing activity to combat coronavirus infections, relative to the large-sized nAbs. In addition, 5-HB and HR121 can mimic the theoretical HR1 groove that only exposed in intermediate state of S and serve as effective immunogens for immunizing camelid/alpaca to obtain nanobodies, as well as baits for screening aptamers that inhibit 6-HB formation. Thus, it is feasible to screen anti-S2 nanobodies or aptamers with potent cross-inhibitory activity against future outbreaks of novel coronaviruses.

S2-based vaccines might also be promising candidates for the design of cross-reactive pan-coronavirus vaccines. However, the relatively low immunogenicity of the S2-based antigens remains a limited factor. N-glycan or other segments of the trimeric S protein may cover the neutralizing epitope, making it difficult to recognize by the immune system. It is important to consider the effects of glycosylation or cryptic epitopes when designing broad-spectrum SARS-CoV-2 vaccines. Therefore, novel adjuvants (e.g., cGAS-STING stimulator) that adequately stimulate immune response to the S2-antigens and different antigen-presentation strategies (e.g., mosaic nanoparticle) that present multi and repeat antigens as well as S2-based vaccine with glycan-site deletion that fully exposes more conserved epitopes should be considered.^165–169^ In this way, the vaccine might be more immunogenic and provide stronger cross-protection.

Overall, the conserved neutralizing elements (the FP, SH, and HR1-HR2 bundle) are promising targets for the design of cross-reactive fusion inhibitors or vaccine candidates to combat the current COVID-19 pandemic as well as the future outbreak of novel human coronaviruses and potential spillover of zoonotic coronaviruses.

## References

[1] Fehr AR, Perlman S (2015). Coronaviruses: an overview of their replication and pathogenesis. Methods Mol. Biol..

[2] Zhou P (2020). A pneumonia outbreak associated with a new coronavirus of probable bat origin. Nature.

[3] Wang Q (2014). Bat origins of MERS-CoV supported by bat coronavirus HKU4 usage of human receptor CD26. Cell Host Microbe.

[4] Zhou Z, Qiu Y, Ge X (2021). The taxonomy, host range and pathogenicity of coronaviruses and other viruses in the Nidovirales order. Anim. Dis..

[5] van der Hoek L (2004). Identification of a new human coronavirus. Nat. Med..

[6] McIntosh K, Becker WB, Chanock RM (1967). Growth in suckling-mouse brain of “IBV-like” viruses from patients with upper respiratory tract disease. Proc. Natl Acad. Sci. USA.

[7] Lau SK (2006). Coronavirus HKU1 and other coronavirus infections in Hong Kong. J. Clin. Microbiol..

[8] Vetterlein W, Hesse R (1965). Electron microscopic picture of viral hepatitis in man and mouse. Arch. Exp. Veterinarmed..

[9] Falsey AR, Walsh EE (2003). Novel coronavirus and severe acute respiratory syndrome. Lancet.

[10] Zaki AM, van Boheemen S, Bestebroer TM, Osterhaus AD, Fouchier RA (2012). Isolation of a novel coronavirus from a man with pneumonia in Saudi Arabia. N. Engl. J. Med..

[11] Zhu N (2020). A Novel Coronavirus from Patients with Pneumonia in China, 2019. N. Engl. J. Med..

[12] Cui J, Li F, Shi ZL (2019). Origin and evolution of pathogenic coronaviruses. Nat. Rev. Microbiol..

[13] Rabaan AA (2020). SARS-CoV-2, SARS-CoV, and MERS-COV: a comparative overview. Infez. Med..

[14] Hu B, Guo H, Zhou P, Shi ZL (2021). Characteristics of SARS-CoV-2 and COVID-19. Nat. Rev. Microbiol..

[15] Ke Z (2020). Structures and distributions of SARS-CoV-2 spike proteins on intact virions. Nature.

[16] Jackson CB, Farzan M, Chen B, Choe H (2022). Mechanisms of SARS-CoV-2 entry into cells. Nat. Rev. Mol. Cell Biol..

[17] Li F (2016). Structure, function, and evolution of coronavirus spike proteins. Annu. Rev. Virol..

[18] Yeung KS, Yamanaka GA, Meanwell NA (2006). Severe acute respiratory syndrome coronavirus entry into host cells: opportunities for therapeutic intervention. Med. Res. Rev..

[19] Letko M, Marzi A, Munster V (2020). Functional assessment of cell entry and receptor usage for SARS-CoV-2 and other lineage B betacoronaviruses. Nat. Microbiol..

[20] Wang Q (2020). Structural and functional basis of SARS-CoV-2 entry by using human ACE2. Cell.

[21] Dai L (2022). Efficacy and safety of the RBD-dimer-based Covid-19 vaccine ZF2001 in adults. N. Engl. J. Med..

[22] Yang S (2021). Safety and immunogenicity of a recombinant tandem-repeat dimeric RBD-based protein subunit vaccine (ZF2001) against COVID-19 in adults: two randomised, double-blind, placebo-controlled, phase 1 and 2 trials. Lancet Infect. Dis..

[23] Yang J (2020). A vaccine targeting the RBD of the S protein of SARS-CoV-2 induces protective immunity. Nature.

[24] Shi R (2020). A human neutralizing antibody targets the receptor-binding site of SARS-CoV-2. Nature.

[25] Han Y (2022). mRNA vaccines expressing homo-prototype/Omicron and hetero-chimeric RBD-dimers against SARS-CoV-2. Cell Res..

[26] Hoffmann M (2021). SARS-CoV-2 variants B.1.351 and P.1 escape from neutralizing antibodies. Cell.

[27] Wang Q (2023). Alarming antibody evasion properties of rising SARS-CoV-2 BQ and XBB subvariants. Cell.

[28] Arora P (2023). Omicron sublineage BQ.1.1 resistance to monoclonal antibodies. Lancet Infect. Dis..

[29] Huang M (2022). Atlas of currently available human neutralizing antibodies against SARS-CoV-2 and escape by Omicron sub-variants BA.1/BA.1.1/BA.2/BA.3. Immunity.

[30] Zhang X (2023). Omicron sublineage recombinant XBB evades neutralising antibodies in recipients of BNT162b2 or CoronaVac vaccines. Lancet Microbe.

[31] K A (2022). Molecular aspects of Omicron, vaccine development, and recombinant strain XE: a review. J. Med. Virol..

[32] Elhazmi A (2021). Severe acute respiratory syndrome coronavirus 2 (SARS-CoV-2) and Middle East Respiratory Syndrome Coronavirus (MERS-CoV) coinfection: a unique case series. Travel Med. Infect. Dis..

[33] Su S, Li W, Jiang S (2022). Developing pan-beta-coronavirus vaccines against emerging SARS-CoV-2 variants of concern. Trends Immunol..

[34] Jiang S, He Y, Liu S (2005). SARS vaccine development. Emerg. Infect. Dis..

[35] Du L, Tai W, Zhou Y, Jiang S (2016). Vaccines for the prevention against the threat of MERS-CoV. Expert Rev. Vaccines.

[36] Temmam S (2022). Bat coronaviruses related to SARS-CoV-2 and infectious for human cells. Nature.

[37] Zhou H (2021). Identification of novel bat coronaviruses sheds light on the evolutionary origins of SARS-CoV-2 and related viruses. Cell.

[38] Ge XY (2013). Isolation and characterization of a bat SARS-like coronavirus that uses the ACE2 receptor. Nature.

[39] Liu K (2021). Binding and molecular basis of the bat coronavirus RaTG13 virus to ACE2 in humans and other species. Cell.

[40] Xu Z (2022). Binding and structural basis of equine ACE2 to RBDs from SARS-CoV, SARS-CoV-2 and related coronaviruses. Nat. Commun..

[41] Menachery VD (2015). A SARS-like cluster of circulating bat coronaviruses shows potential for human emergence. Nat. Med..

[42] Shah VK, Firmal P, Alam A, Ganguly D, Chattopadhyay S (2020). Overview of immune response during SARS-CoV-2 infection: lessons from the past. Front Immunol..

[43] Wrapp D (2020). Cryo-EM structure of the 2019-nCoV spike in the prefusion conformation. Science.

[44] Cai Y (2020). Distinct conformational states of SARS-CoV-2 spike protein. Science.

[45] Tai L (2021). Nanometer-resolution in situ structure of the SARS-CoV-2 postfusion spike protein. Proc. Natl Acad. Sci. USA.

[46] Connolly SA, Jardetzky TS, Longnecker R (2021). The structural basis of herpesvirus entry. Nat. Rev. Microbiol..

[47] Hoffmann M, Kleine-Weber H, Pohlmann S (2020). A multibasic cleavage site in the spike protein of SARS-CoV-2 is essential for infection of human lung cells. Mol. Cell.

[48] Yu S (2022). SARS-CoV-2 spike engagement of ACE2 primes S2’ site cleavage and fusion initiation. Proc. Natl Acad. Sci. USA.

[49] Lin X (2022). An engineered 5-helix bundle derived from SARS-CoV-2 S2 pre-binds sarbecoviral spike at both serological- and endosomal-pH to inhibit virus entry. Emerg. Microbes Infect..

[50] Yang K (2022). Structural conservation among variants of the SARS-CoV-2 spike postfusion bundle. Proc. Natl Acad. Sci. USA.

[51] Dacon C (2022). Broadly neutralizing antibodies target the coronavirus fusion peptide. Science.

[52] Low JS (2022). ACE2-binding exposes the SARS-CoV-2 fusion peptide to broadly neutralizing coronavirus antibodies. Science.

[53] Sun X (2022). Neutralization mechanism of a human antibody with pan-coronavirus reactivity including SARS-CoV-2. Nat. Microbiol..

[54] Bianchini F (2023). Human neutralizing antibodies to cold linear epitopes and subdomain 1 of the SARS-CoV-2 spike glycoprotein. Sci. Immunol..

[55] Pinto D (2021). Broad betacoronavirus neutralization by a stem helix-specific human antibody. Science.

[56] Zhou P (2022). A human antibody reveals a conserved site on beta-coronavirus spike proteins and confers protection against SARS-CoV-2 infection. Sci. Transl. Med..

[57] Hurlburt NK (2022). Structural definition of a pan-sarbecovirus neutralizing epitope on the spike S2 subunit. Commun. Biol..

[58] Zhou P (2023). Broadly neutralizing anti-S2 antibodies protect against all three human betacoronaviruses that cause deadly disease. Immunity.

[59] Shi W (2022). Vaccine-elicited murine antibody WS6 neutralizes diverse beta-coronaviruses by recognizing a helical stem supersite of vulnerability. Structure.

[60] Sauer MM (2021). Structural basis for broad coronavirus neutralization. Nat. Struct. Mol. Biol..

[61] Wang C (2021). A conserved immunogenic and vulnerable site on the coronavirus spike protein delineated by cross-reactive monoclonal antibodies. Nat. Commun..

[62] Hsieh CL (2021). Stabilized coronavirus spike stem elicits a broadly protective antibody. Cell Rep..

[63] Li T (2021). A novel linear and broadly neutralizing peptide in the SARS-CoV-2 S2 protein for universal vaccine development. Cell Mol. Immunol..

[64] Dai L, Gao GF (2021). Viral targets for vaccines against COVID-19. Nat. Rev. Immunol..

[65] Li Y (2020). Linear epitopes of SARS-CoV-2 spike protein elicit neutralizing antibodies in COVID-19 patients. Cell Mol. Immunol..

[66] Ladner JT (2021). Epitope-resolved profiling of the SARS-CoV-2 antibody response identifies cross-reactivity with endemic human coronaviruses. Cell Rep. Med..

[67] Li Y (2021). Linear epitope landscape of the SARS-CoV-2 Spike protein constructed from 1,051 COVID-19 patients. Cell Rep..

[68] Xiong Q (2022). Close relatives of MERS-CoV in bats use ACE2 as their functional receptors. Nature.

[69] Jiang S, Lin K, Strick N, Neurath AR (1993). HIV-1 inhibition by a peptide. Nature.

[70] Liu S (2004). Interaction between heptad repeat 1 and 2 regions in spike protein of SARS-associated coronavirus: implications for virus fusogenic mechanism and identification of fusion inhibitors. Lancet.

[71] Lu L (2014). Structure-based discovery of Middle East respiratory syndrome coronavirus fusion inhibitor. Nat. Commun..

[72] Xia S (2020). Fusion mechanism of 2019-nCoV and fusion inhibitors targeting HR1 domain in spike protein. Cell Mol. Immunol..

[73] Gao J (2013). Structure of the fusion core and inhibition of fusion by a heptad repeat peptide derived from the S protein of Middle East respiratory syndrome coronavirus. J. Virol..

[74] Wang X, Xia S, Zhu Y, Lu L, Jiang S (2021). Pan-coronavirus fusion inhibitors as the hope for today and tomorrow. Protein Cell.

[75] Xia S (2019). A pan-coronavirus fusion inhibitor targeting the HR1 domain of human coronavirus spike. Sci. Adv..

[76] Xia S (2020). Inhibition of SARS-CoV-2 (previously 2019-nCoV) infection by a highly potent pan-coronavirus fusion inhibitor targeting its spike protein that harbors a high capacity to mediate membrane fusion. Cell Res..

[77] Xia S (2022). Peptide-based pan-CoV fusion inhibitors maintain high potency against SARS-CoV-2 Omicron variant. Cell Res..

[78] Xia S (2021). Structural and functional basis for pan-CoV fusion inhibitors against SARS-CoV-2 and its variants with preclinical evaluation. Signal Transduct. Target Ther..

[79] Yan F, Gao F (2022). EK1 with dual Q1004E/N1006I mutation: a promising fusion inhibitor for the HR1 domain of SARS-CoV-2. J. Infect..

[80] Yang K (2022). Nanomolar inhibition of SARS-CoV-2 infection by an unmodified peptide targeting the prehairpin intermediate of the spike protein. Proc. Natl Acad. Sci. USA.

[81] Sun H (2020). Structural basis of HCoV-19 fusion core and an effective inhibition peptide against virus entry. Emerg. Microbes Infect..

[82] Wild CT, Shugars DC, Greenwell TK, McDanal CB, Matthews TJ (1994). Peptides corresponding to a predictive alpha-helical domain of human immunodeficiency virus type 1 gp41 are potent inhibitors of virus infection. Proc. Natl Acad. Sci. USA.

[83] Root MJ, Kay MS, Kim PS (2001). Protein design of an HIV-1 entry inhibitor. Science.

[84] Hollmann A, Matos PM, Augusto MT, Castanho MA, Santos NC (2013). Conjugation of cholesterol to HIV-1 fusion inhibitor C34 increases peptide-membrane interactions potentiating its action. PLoS ONE.

[85] Park JE, Gallagher T (2017). Lipidation increases antiviral activities of coronavirus fusion-inhibiting peptides. Virology.

[86] Zhu Y (2021). SARS-CoV-2-derived fusion inhibitor lipopeptides exhibit highly potent and broad-spectrum activity against divergent human coronaviruses. Signal Transduct. Target Ther..

[87] de Vries RD (2021). Intranasal fusion inhibitory lipopeptide prevents direct-contact SARS-CoV-2 transmission in ferrets. Science.

[88] Garay RP, El-Gewely R, Armstrong JK, Garratty G, Richette P (2012). Antibodies against polyethylene glycol in healthy subjects and in patients treated with PEG-conjugated agents. Expert Opin. Drug Deliv..

[89] Zhou J (2022). A highly potent and stable pan-coronavirus fusion inhibitor as a candidate prophylactic and therapeutic for COVID-19 and other coronavirus diseases. Acta Pharm. Sin. B.

[90] Salmon-Ceron D (2010). Immunogenicity and safety of an HIV-1 lipopeptide vaccine in healthy adults: a phase 2 placebo-controlled ANRS trial. AIDS.

[91] Zang R (2020). Cholesterol 25-hydroxylase suppresses SARS-CoV-2 replication by blocking membrane fusion. Proc. Natl Acad. Sci. USA.

[92] Wang S (2020). Cholesterol 25-Hydroxylase inhibits SARS-CoV-2 and other coronaviruses by depleting membrane cholesterol. EMBO J..

[93] Lan Q (2022). A palmitic acid-conjugated, peptide-based pan-CoV fusion inhibitor potently inhibits infection of SARS-CoV-2 Omicron and other variants of concern. Viruses.

[94] Lan Q (2021). 25-Hydroxycholesterol-conjugated EK1 peptide with potent and broad-spectrum inhibitory activity against SARS-CoV-2, its variants of concern, and other human coronaviruses. Int J. Mol. Sci..

[95] Xing L (2022). A five-helix-based SARS-CoV-2 fusion inhibitor targeting heptad repeat 2 domain against SARS-CoV-2 and its variants of concern. Viruses.

[96] Bi W, Chen G, Dang B (2022). Novel engineered SARS-CoV-2 HR1 trimer exhibits improved potency and broad-spectrum activity against SARS-CoV-2 and its variants. J. Virol..

[97] Sun Y, Zhang H, Shi J, Zhang Z, Gong R (2017). Identification of a novel inhibitor against Middle East respiratory syndrome coronavirus. Viruses.

[98] Lusvarghi S, Bewley CA (2016). Griffithsin: an antiviral lectin with outstanding therapeutic potential. Viruses.

[99] Cai Y (2020). Griffithsin with a broad-spectrum antiviral activity by binding glycans in viral glycoprotein exhibits strong synergistic effect in combination with a pan-coronavirus fusion inhibitor targeting SARS-CoV-2 spike S2 subunit. Virol. Sin..

[100] O’Keefe BR (2010). Broad-spectrum in vitro activity and in vivo efficacy of the antiviral protein griffithsin against emerging viruses of the family Coronaviridae. J. Virol..

[101] Cai Y (2021). A bivalent protein targeting glycans and HR1 domain in spike protein potently inhibited infection of SARS-CoV-2 and other human coronaviruses. Cell Biosci..

[102] Duan Q (2022). A modified fibronectin type III domain-conjugated, long-acting pan-coronavirus fusion inhibitor with extended half-life. Viruses.

[103] Yin W (2020). Structural basis for inhibition of the RNA-dependent RNA polymerase from SARS-CoV-2 by remdesivir. Science.

[104] Kabinger F (2021). Mechanism of molnupiravir-induced SARS-CoV-2 mutagenesis. Nat. Struct. Mol. Biol..

[105] Lei S, Chen X, Wu J, Duan X, Men K (2022). Small molecules in the treatment of COVID-19. Signal Transduct. Target Ther..

[106] Pu J (2022). The analogs of furanyl methylidene rhodanine exhibit broad-spectrum inhibitory and inactivating activities against enveloped viruses, including SARS-CoV-2 and its variants. Viruses.

[107] Yang C (2020). Salvianolic acid C potently inhibits SARS-CoV-2 infection by blocking the formation of six-helix bundle core of spike protein. Signal Transduct. Target Ther..

[108] Gentile D (2022). Targeting the SARS-CoV-2 HR1 with small molecules as inhibitors of the fusion process. Int. J. Mol. Sci..

[109] Villalain J (2010). Membranotropic effects of arbidol, a broad anti-viral molecule, on phospholipid model membranes. J. Phys. Chem. B.

[110] Kadam RU, Wilson IA (2017). Structural basis of influenza virus fusion inhibition by the antiviral drug Arbidol. Proc. Natl Acad. Sci. USA.

[111] Ford N (2020). Systematic review of the efficacy and safety of antiretroviral drugs against SARS, MERS or COVID-19: initial assessment. J. Int. AIDS Soc..

[112] Zhao H (2021). Cross-linking peptide and repurposed drugs inhibit both entry pathways of SARS-CoV-2. Nat. Commun..

[113] Vankadari N (2020). Arbidol: a potential antiviral drug for the treatment of SARS-CoV-2 by blocking trimerization of the spike glycoprotein. Int. J. Antimicrob. Agents.

[114] Wang X (2020). The anti-influenza virus drug, arbidol is an efficient inhibitor of SARS-CoV-2 in vitro. Cell Discov..

[115] Zhang JN (2020). Potential of arbidol for post-exposure prophylaxis of COVID-19 transmission: a preliminary report of a retrospective cohort study. Curr. Med. Sci..

[116] Zhao J (2022). A trial of arbidol hydrochloride in adults with COVID-19. Chin. Med. J..

[117] Deng L (2020). Arbidol combined with LPV/r versus LPV/r alone against Corona Virus Disease 2019: a retrospective cohort study. J. Infect..

[118] Lian N (2020). Umifenovir treatment is not associated with improved outcomes in patients with coronavirus disease 2019: a retrospective study. Clin. Microbiol. Infect..

[119] Jana ID (2022). Targeting an evolutionarily conserved “E-L-L” motif in the spike protein to develop a small molecule fusion inhibitor against SARS-CoV-2. PNAS Nexus.

[120] Yang C (2021). Drug repurposing of itraconazole and estradiol benzoate against COVID-19 by blocking SARS-CoV-2 spike protein-mediated membrane fusion. Adv. Ther..

[121] Mondal S, Karmakar A, Mallick T, Begum NA (2021). Exploring the efficacy of naturally occurring biflavone based antioxidants towards the inhibition of the SARS-CoV-2 spike glycoprotein mediated membrane fusion. Virology.

[122] Yin X, Chen L, Yuan S, Liu L, Gao Z (2021). A robust high-throughput fluorescent polarization assay for the evaluation and screening of SARS-CoV-2 fusion inhibitors. Bioorg. Chem..

[123] Frey G (2006). Small molecules that bind the inner core of gp41 and inhibit HIV envelope-mediated fusion. Proc. Natl Acad. Sci. USA.

[124] Cao Y (2022). BA.2.12.1, BA.4 and BA.5 escape antibodies elicited by Omicron infection. Nature.

[125] Dejnirattisai W (2022). SARS-CoV-2 Omicron-B.1.1.529 leads to widespread escape from neutralizing antibody responses. Cell.

[126] Iketani S (2022). Antibody evasion properties of SARS-CoV-2 Omicron sublineages. Nature.

[127] Okba NMA (2020). Severe acute respiratory syndrome coronavirus 2-specific antibody responses in coronavirus disease patients. Emerg. Infect. Dis..

[128] Chai M (2022). A high-throughput single cell-based antibody discovery approach against the full-length SARS-CoV-2 spike protein suggests a lack of neutralizing antibodies targeting the highly conserved S2 domain. Brief. Bioinform..

[129] Ng KW (2022). SARS-CoV-2 S2-targeted vaccination elicits broadly neutralizing antibodies. Sci. Transl. Med..

[130] Halfmann PJ (2022). Multivalent S2-based vaccines provide broad protection against SARS-CoV-2 variants of concern and pangolin coronaviruses. EBioMedicine.

[131] Xu K (2018). Epitope-based vaccine design yields fusion peptide-directed antibodies that neutralize diverse strains of HIV-1. Nat. Med..

[132] Nachbagauer R (2021). A chimeric hemagglutinin-based universal influenza virus vaccine approach induces broad and long-lasting immunity in a randomized, placebo-controlled phase I trial. Nat. Med..

[133] Belouzard S, Chu VC, Whittaker GR (2009). Activation of the SARS coronavirus spike protein via sequential proteolytic cleavage at two distinct sites. Proc. Natl Acad. Sci. USA.

[134] Madu IG, Roth SL, Belouzard S, Whittaker GR (2009). Characterization of a highly conserved domain within the severe acute respiratory syndrome coronavirus spike protein S2 domain with characteristics of a viral fusion peptide. J. Virol..

[135] Maeda D (2021). Killed whole-genome reduced-bacteria surface-expressed coronavirus fusion peptide vaccines protect against disease in a porcine model. Proc. Natl Acad. Sci. USA.

[136] Walls AC (2020). Structure, function, and antigenicity of the SARS-CoV-2 spike glycoprotein. Cell.

[137] Low JS (2021). Clonal analysis of immunodominance and cross-reactivity of the CD4 T cell response to SARS-CoV-2. Science.

[138] Yuan Y (2017). Cryo-EM structures of MERS-CoV and SARS-CoV spike glycoproteins reveal the dynamic receptor binding domains. Nat. Commun..

[139] Ma X (2020). Nanoparticle vaccines based on the receptor binding domain (RBD) and heptad repeat (HR) of SARS-CoV-2 elicit robust protective immune responses. Immunity.

[140] He C (2022). A self-assembled trimeric protein vaccine induces protective immunity against Omicron variant. Nat. Commun..

[141] Pang W (2022). A variant-proof SARS-CoV-2 vaccine targeting HR1 domain in S2 subunit of spike protein. Cell Res..

[142] Sette A, Crotty S (2021). Adaptive immunity to SARS-CoV-2 and COVID-19. Cell.

[143] Ni L (2020). Detection of SARS-CoV-2-specific humoral and cellular immunity in COVID-19 convalescent individuals. Immunity.

[144] Altmann DM, Boyton RJ (2020). SARS-CoV-2 T cell immunity: Specificity, function, durability, and role in protection. Sci. Immunol..

[145] Kinoshita H (2021). Robust antibody and T cell responses to SARS-CoV-2 in patients with antibody deficiency. J. Clin. Immunol..

[146] Ng OW (2016). Memory T cell responses targeting the SARS coronavirus persist up to 11 years post-infection. Vaccine.

[147] Le Bert N (2020). SARS-CoV-2-specific T cell immunity in cases of COVID-19 and SARS, and uninfected controls. Nature.

[148] Zhang Z (2022). Humoral and cellular immune memory to four COVID-19 vaccines. Cell.

[149] Peng Y (2020). Broad and strong memory CD4(+) and CD8(+) T cells induced by SARS-CoV-2 in UK convalescent individuals following COVID-19. Nat. Immunol..

[150] Kalimuddin S (2021). Early T cell and binding antibody responses are associated with COVID-19 RNA vaccine efficacy onset. Med.

[151] Chandrashekar A (2022). Vaccine protection against the SARS-CoV-2 Omicron variant in macaques. Cell.

[152] Tan AT (2021). Early induction of functional SARS-CoV-2-specific T cells associates with rapid viral clearance and mild disease in COVID-19 patients. Cell Rep..

[153] Sekine T (2020). Robust T cell immunity in convalescent individuals with asymptomatic or mild COVID-19. Cell.

[154] Grifoni A (2020). Targets of T cell responses to SARS-CoV-2 coronavirus in humans with COVID-19 disease and unexposed individuals. Cell.

[155] Braun J (2020). SARS-CoV-2-reactive T cells in healthy donors and patients with COVID-19. Nature.

[156] Tarke A (2021). Impact of SARS-CoV-2 variants on the total CD4(+) and CD8(+) T cell reactivity in infected or vaccinated individuals. Cell Rep. Med..

[157] Tarke A (2022). SARS-CoV-2 vaccination induces immunological T cell memory able to cross-recognize variants from Alpha to Omicron. Cell.

[158] Gao F (2023). Spheromers reveal robust T cell responses to the Pfizer/BioNTech vaccine and attenuated peripheral CD8(+) T cell responses post SARS-CoV-2 infection. Immunity.

[159] Muyldermans S (2013). Nanobodies: natural single-domain antibodies. Annu. Rev. Biochem..

[160] Zhou J, Rossi J (2017). Aptamers as targeted therapeutics: current potential and challenges. Nat. Rev. Drug Discov..

[161] Xu J (2021). Nanobodies from camelid mice and llamas neutralize SARS-CoV-2 variants. Nature.

[162] Yang J (2022). A potent neutralizing nanobody targeting the spike receptor-binding domain of SARS-CoV-2 and the structural basis of its intimate binding. Front. Immunol..

[163] Liu X (2021). Neutralizing Aptamers block S/RBD-ACE2 interactions and prevent host cell infection. Angew. Chem. Int Ed. Engl..

[164] Sun M (2021). Aptamer blocking strategy inhibits SARS-CoV-2 virus infection. Angew. Chem. Int. Ed. Engl..

[165] Watanabe Y, Allen JD, Wrapp D, McLellan JS, Crispin M (2020). Site-specific glycan analysis of the SARS-CoV-2 spike. Science.

[166] Huang HY (2022). Vaccination with SARS-CoV-2 spike protein lacking glycan shields elicits enhanced protective responses in animal models. Sci. Transl. Med..

[167] Wu CY (2022). Glycosite-deleted mRNA of SARS-CoV-2 spike protein as a broad-spectrum vaccine. Proc. Natl Acad. Sci. USA.

[168] Ma C (2021). A novel inactivated whole-cell Pseudomonas aeruginosa vaccine that acts through the cGAS-STING pathway. Signal Transduct. Target Ther..

[169] Cohen AA (2021). Mosaic nanoparticles elicit cross-reactive immune responses to zoonotic coronaviruses in mice. Science.

